# Root-associated endophytic bacterial community composition and structure of three medicinal licorices and their changes with the growing year

**DOI:** 10.1186/s12866-020-01977-3

**Published:** 2020-09-21

**Authors:** Hanli Dang, Tao Zhang, Guifang Li, Yudi Mu, Xinhua Lv, Zhongke Wang, Li Zhuang

**Affiliations:** 1 College of Life Sciences, Key Laboratory of Xinjiang Phytomedicine Resource Utilization, Ministry of Education, Shihezi University, Shihezi, 832003 Xinjiang China; 2 1300 UNIVERSITY Ave, Madison, WI 53706 USA

**Keywords:** Endophytic bacteria, Growing year, High-throughput sequencing, Liquorice species, Plant secondary metabolites, Soil physicochemical

## Abstract

**Background:**

The dried roots and rhizomes of medicinal licorices are widely used worldwide as a traditional medicinal herb, which are mainly attributed to a variety of bioactive compounds that can be extracted from licorice root. Endophytes and plants form a symbiotic relationship, which is an important source of host secondary metabolites.

**Results:**

In this study, we used high-throughput sequencing technology and high-performance liquid chromatography to explore the composition and structure of the endophytic bacterial community and the content of bioactive compounds (glycyrrhizic acid, liquiritin and total flavonoids) in different species of medicinal licorices (*Glycyrrhiza uralensis*, *Glycyrrhiza glabra*, and *Glycyrrhiza inflata*) and in different planting years (1–3 years). Our results showed that the contents of the bioactive compounds in the roots of medicinal licorices were not affected by the species, but were significantly affected by the main effect growing year (1–3) (*P* < 0.05), and with a trend of stable increase in the contents observed with each growing year. In 27 samples, a total of 1,979,531 effective sequences were obtained after quality control, and 2432 effective operational taxonomic units (OTUs) were obtained at 97% identity. The phylum Proteobacteria, Actinobacteria, Bacteroidetes and Firmicutes, and the genera unified-*Rhizobiaceae*, *Pseudomonas*, *Novosphingobium*, and *Pantoea* were significantly dominant in the 27 samples. Distance-based redundancy analysis (db-RDA) showed that the content of total flavonoids explained the differences in composition and distribution of endophytic bacterial communities in roots of cultivated medicinal liquorices to the greatest extent. Total soil salt was the most important factor that significantly affected the endophytic bacterial community in soil factors, followed by ammonium nitrogen and nitrate nitrogen. Among the leaf nutrition factors, leaf water content had the most significant effect on the endophytic bacterial community, followed by total phosphorus and total potassium.

**Conclusions:**

This study not only provides information on the composition and distribution of endophytic bacteria in the roots of medicinal licorices, but also reveals the influence of abiotic factors on the community of endophytic bacteria and bioactive compounds, which provides a reference for improving the quality of licorice.

## Background

The term endophyte describes those taxa that can live within plant tissues, either within or between host cells [1]. These endophytes include bacteria, fungi, archaea and unicellular eukaryotes [2]. The internal niches of plants provide a protective barrier from environmental influences that allow bacteria to live and reproduce [3]. In return, plants may benefit from endophytic associations. First, endophytic bacteria can promote the growth and development of host plants by producing a range of nutrients and promoting primary and secondary nutrient uptake through atmospheric nitrogen fixation [4]. Second, endophytic bacteria can participate in aspects of the plant’s phosphate solubilization activity [5], such as osmotic adjustment [6] and stomatal regulation [7], to increase the plant’s ecological adaptability. Furthermore, endophytic bacteria may overcome environmental stress conditions, such as drought and soil salinity stress [8, 9], and improve plant growth. Also as biological control agents, endophytic bacteria can produce or promote the production of secondary metabolites by host plants to reduce or prevent damage caused by certain pathogens [10, 11]. Numerous studies have shown that an in-depth understanding of the diversity of endophytic bacteria will elucidate the function of the interaction between microorganisms and plants, which will be conducive to the development of strategies for ecological environment restoration and sustainable agricultural, such as remediation of the soil environment [12] and increasing crop production [13].

Endophytic bacteria have been isolated from both monocotyledonous and dicotyledonous plants [3], ranging from woody plants [14], such as walnut and poplar, to herbaceous crop plants, such as rice and wheat. Modern molecular technology, especially high-throughput sequencing technology, has facilitated characterization of the composition and structure of microbial communities in different plants and has substantially increased our understanding of the composition and diversity of endophytic bacterial communities in the plants [6, 15]. These techniques provide information more rapidly and accurately than conventional culturing methods, which are time-consuming and may not be applicable for microorganisms that cannot be cultured [16, 17]. Although high-throughput sequencing technology is still subject to several challenges in terms of analysis and visualization of genomic data that relies heavily on the performance of automated pipelines [18], these techniques can be applied to comprehensive analyses of plant tissues RNA and DNA sequencing and are within the reach of most laboratories. High-throughput sequencing based on the targeted phylogenetic marker 16S rRNA can be used to effectively characterize the diversity of microbial communities [19], which makes it a practical approach to the isolation and identification of the community composition and diversity of endogenous bacteria in medicinal licorices.

The dried roots and rhizomes of three medicinal licorices (*Glycyrrhiza uralensis*, *Glycyrrhiza inflata*, and *Glycyrrhiza glabra*) are widely used worldwide as a traditional medicinal herb [20]. Many clinical and experimental studies have demonstrated that the root of medicinal licorices has a variety of pharmacological properties [21], such as anti-inflammatory, antiviral and antitumor, immunomodulatory, hepatoprotective activities, which are attributed to a variety of bioactive constituents extracted from licorice root, mainly including triterpene saponins and flavonoids [22]. Glycyrrhizic acid, which is present at the highest levels among the triterpene saponins, is an important pharmacologically bioactive constituent with anti-inflammatory [23], antiviral, immune regulation and other biological properties. Recent studies have shown that licorice flavonoids have a variety of pharmacological activities and have gradually become a hot spot in pharmacological research [24, 25].

Bioactive compounds contained within medicinal plants are the material basis for clinical efficacy and an important index of the quality of medicinal materials. Furthermore, these materials are the evolutionary result of the interaction between plants and their natural environment [26, 27]. Studies have shown that the accumulation of bioactive compounds in licorice roots is affected by both the ecological environment and regulation of nutrient elements, such as nitrogen sources and phosphate concentrations in the soil [28], to patterns of distribution in the root distribution pattern that are affected by factors such as year [29] and biomass [30].

Currently, there is little information about the composition of endophytic bacterial communities in the roots of medicinal licorices at different times in the planting year, and the factors influencing the community structure are not clear. Furthermore, the factors influencing the content of bioactive compounds of medicinal licorices also remain to be elucidated. Therefore, in this study, we conducted a three-year licorice planting experiment in the field and used Illumina high-throughput sequencing technology to detect the composition and diversity of the endophytic bacterial community of medicinal licorice roots to explore the factors affecting the accumulation of bioactive compounds and the structure of the endophytic bacterial community.

## Results

### The effect of the growing year on the content of bioactive compounds in the roots of medicinal licorices

**Fig. 1:**
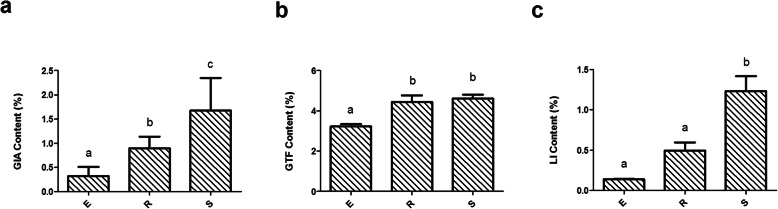
Effect of main effect growing year on the bioactive compounds of licorice roots Description: Ordinate is the content of GIA (**a**), GTF (**b**) and LI (**c**); abscissa is the group name (E, R and S: years 1, 2, and 3, respectively). Different letters indicated statistically significant difference (*P* < 0.05).

The results of two-way ANOVA showed that the contents of the bioactive compounds (glycyrrhizic acid (GIA), liquiritin (LI) and total flavonoid (GTF)) were not significantly affected by the interaction effect between growing year (1–3) and plant species (*Glycyrrhiza uralensis*, *Glycyrrhiza inflata*, and *Glycyrrhiza glabra*) (*P* > 0.05) (Table 1). However, the contents of the bioactive compounds were significantly affected by the main effect growing year (1–3) (*P* < 0.05) (Table 1 and Fig. 1), with a trend of stable increase in the contents observed with each growing year. As shown in Fig. 1, the contents of GIA, LI and GTF in the year 3 were significantly higher than those in year 1, the contents of GIA and GTF in year 2 were significantly higher than those in year 1, and the contents of GIA and LI in year 3 were significantly higher than those in year 2.

**Table 1:** Effect of species and year on the bioactive compounds of licorice root

Source		Type III Sum of Squares	Degrees of freedom	Mean Square	F	***p*** value
**Years**	GIA	8.307	2	4.153	20.087	0.000
	GTF	10.381	2	5.191	97.654	0.000
	LI	5.605	2	2.802	19.053	0.000
**Species**	GIA	0.231	2	0.116	0.559	0.581
	GTF	0.244	2	0.122	2.296	0.129
	LI	0.297	2	0.149	1.010	0.384
Years * Species	GIA	0.398	4	0.099	0.481	0.750
	GTF	0.055	4	0.014	0.260	0.900
	LI	0.284	4	0.071	0.483	0.748

Description: *P* < 0.05 indicates statistical significance

**Fig. 2:**
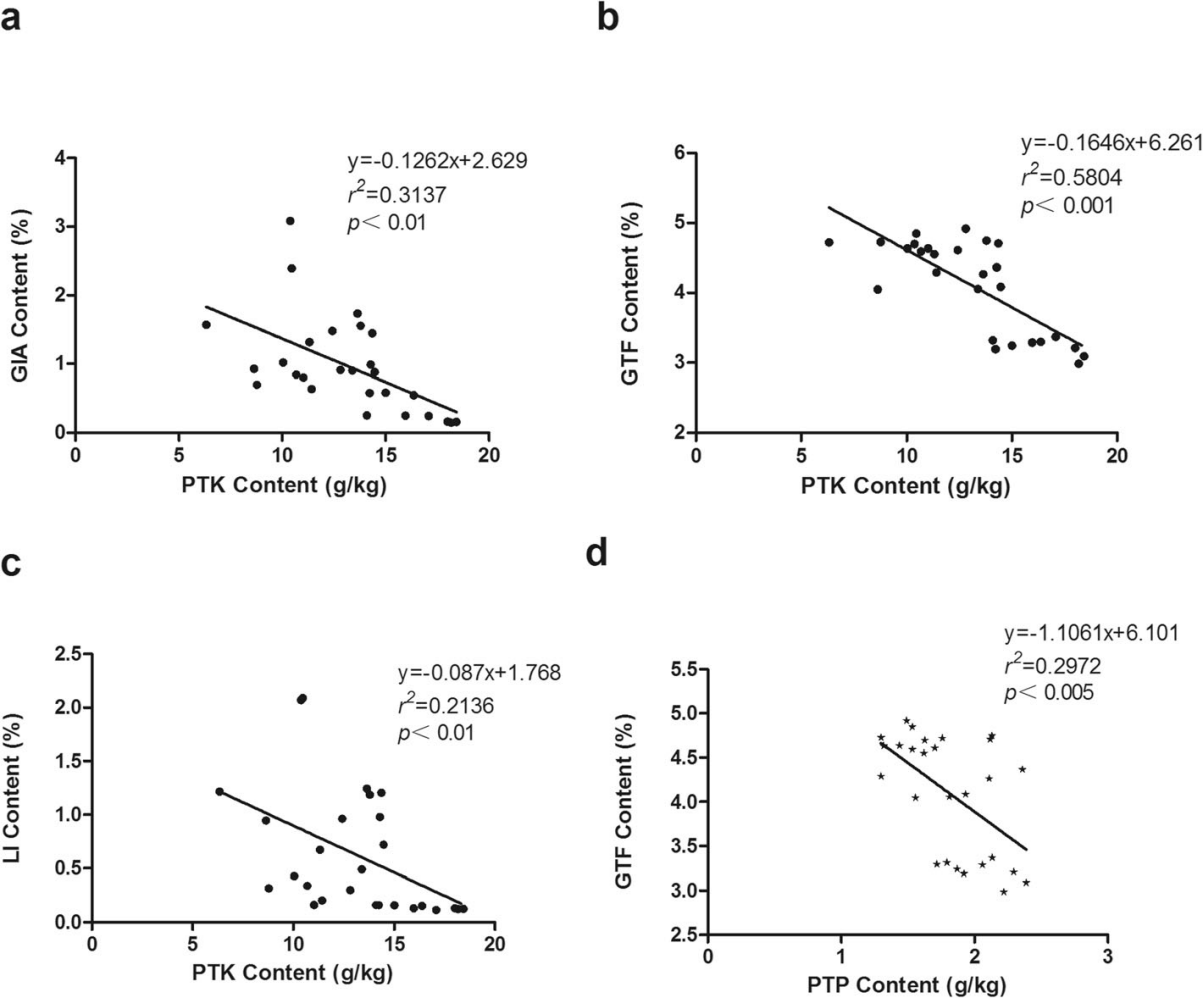
Stepwise multiple linear regression model Description: The content of PTK (**a**, **b**, **c**) and PTP (**d**) were used as independent variables, and the content of GIA, GTF and LI were used as dependent variables.

Details of the temperature, rainfall, leaves and soil factors during the experimental period are presented in Supplementary Table S1. In addition, Spearman correlation analysis showed that the content of bioactive compounds was significantly correlated with soil physicochemical properties and nutritional components of leaves (Table 2). In terms of soil physicochemical factors, GIA and LI had a very significant positive correlation with soil ammonium nitrogen (SAN) (*R*^*2*^ > 0; *P* < 0.01), but had a very significant negative correlation with total salt (TS) (*R*^*2*^ < 0; *P* < 0.01); GTF had a very significant positive correlation with SAN (*R*^*2*^ > 0; *P* < 0.01), but had a very significant negative correlation with nitrate nitrogen (SNN) (*R*^*2*^ < 0; *P* < 0.01), and had a significant negative correlation with TS (*R*^*2*^ < 0.01; *P* < 0.05). In terms of leaf nutrition (Table 2), GIA, LI and GTF were very negatively correlated with water content (PWC), total potassium (PTK) (Fig. 2 a, b, c) and crude fiber (CF). In addition, GTF also showed a very significant negative correlation with total phosphorus (PTP) (Fig. 2d). There was a significant relationship between leaf nutrients and a small number of variables. Specifically, PWC was positively correlated with SNN, but negatively correlated with SAN; organic carbon (POC) was positively correlated with soil total potassium (STK); total nitrogen (PTN) was positively correlated with POC; total phosphorus (PTP) was very positively correlated with SNN, TS and PTN; PTK was very positively correlated with SNN, TS, PWC, PTN and PTP, but negatively correlated with SAN; CF was very positively correlated with PWC and PTK.

**Table 2:** Spearman correlation coefficient of the content of bioactive compounds with soil physicochemical properties and nutritional components of leaves

	GIA	GTF	LI	SOM	STN	STP	STK	SNN	SAN	TS	PWC	POC	PTN	PTP	PTK	CF
GIA	1.000															
GTF	0.774**	1.000														
LI	0.964**	0.716**	1.000													
SOM	0.173	0.156	0.173	1.000												
STN	0.262	0.060	0.233	0.578**	1.000											
STP	0.230	0.032	0.218	0.438**	0.713**	1.000										
STK	− 0.283	−0.025	− 0.283	0.147	− 0.051	− 0.131	1.000									
SNN	−0.363	− 0.490**	− 0.359	0.057	− 0.179	− 0.216	− 0.234	1.000								
SAN	0.600**	0.535**	0.593**	0.396*	0.323	0.433*	0.027	−0.233	1.000							
TS	−0.511**	− 0.449*	− 0.499**	−0.294	− 0.474*	−0.452*	− 0.259	0.760**	− 0.490**	1.000						
PWC	− 731**	−0.807**	−0.737**	0.077	0.019	−0.017	0.073	0.484*	−0.405*	0.343	1.000					
POC	−0.331	−0.114	− 0.311	0.321	0.013	−0.142	0.430*	0.321	0.077	0.324	0.111	1.000				
PTN	−0.261	−0.297	−0.192	0.098	0.101	0.136	0.028	0.243	0.059	0.206	0.245	0.444*	1.000			
PTP	−0.298	−0.537**	−0.223	− 0.073	−0.027	− 0.095	−0.357	0.654**	−0.264	0.656**	0.368	0.259	0.608**	1.000		
PTK	−0.670**	−0.707**	− 0.634**	−0.074	− 0.013	0.048	− 0.123	0.528**	− 0.421*	0.623**	0.644**	0.336	0.587**	0.792**	1.000	
CF	−0.684**	−0.662**	− 0.699**	−0.053	0.070	0.259	0.062	0.207	−0.349	0.173	0.806**	−0.024	0.378	0.227	0.601**	1.000

Description: the values are the correlation coefficients. ** means *P* < 0.01; * means *P* < 0.05

### Composition of bacterial community in the root of medicinal liquorices

**Fig. 3:**
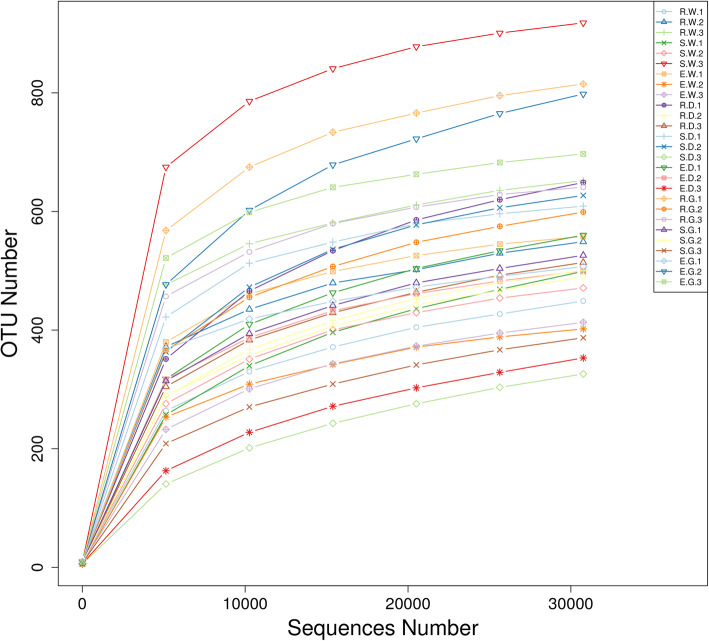
Rarefaction curves of bacterial community composition in 27 samples Description: The rarefaction curves different colors represent different samples (E, R and S: years 1, 2, and 3, respectively; W, G and D: *Glycyrrhiza uralensis*, *Glycyrrhiza glabra*, and *Glycyrrhiza inflata*, respectively; the third number representing the replicate number).

**Fig. 4:**
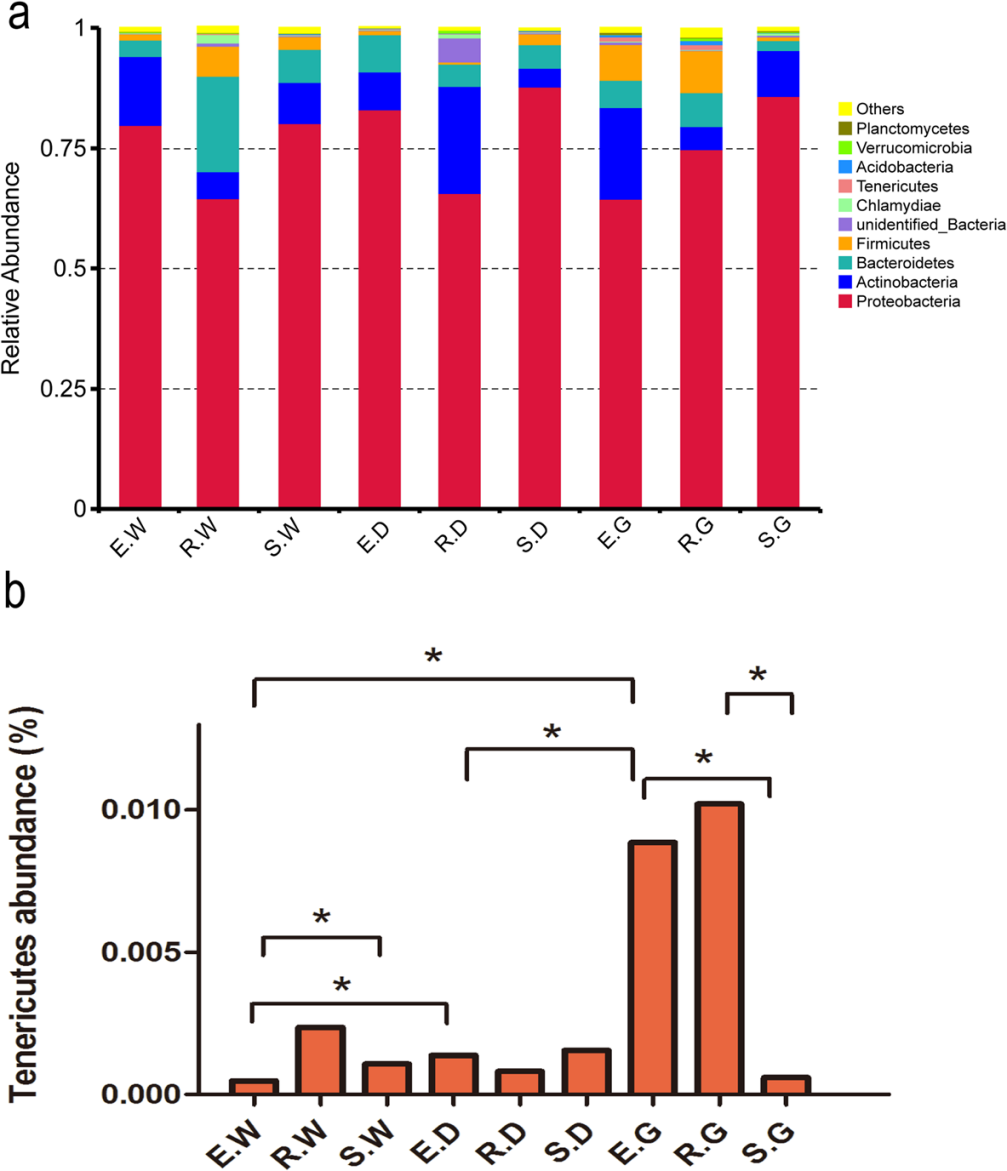
Histograms of relative abundance of the top 10 bacteria at the phyla level of taxonomy (**a**), and significantly different species in the sample (**b**). Description: Ordinate is the relative abundance of bacteria phyla; others refers to are sequences with less or not be annotated; abscissa is the group name (E, R and S: years 1, 2, and 3, respectively; W, G and D: *Glycyrrhiza uralensis*, *Glycyrrhiza glabra*, and *Glycyrrhiza inflata*, respectively). The mark * is significance test *p* < 0.05.

In 27 samples, a total of 1,979,531 effective sequences were obtained after quality control. The sequencing results for each sample are listed in Supplementary Table S2. The effective sequences were clustered into operational taxonomic units (OTUs) with 97% identity, and a total of 2432 OTUs were obtained. The rarefaction curves were normalized to the lowest number of sequences, showed that the number of OTU in each sample increased gradually with quantity of sequence, thus confirming that the amount of sequencing data is adequate (Fig. 3). According to the OTUs, 28 phyla, 45 classes, 106 orders, 216 families, 508 genera and 313 species were annotated. Figure 4a shows the 10 bacterial phyla with the greatest abundance in the bacterial community in the roots of medicinal licorices. Proteobacteria dominated the observed sequences at the phylum level, representing 79.917, 64.420, 80.265, 82.848, 65.733, 87.886, 64.544, 75.024 and 85.847% of the total number of species in E.W, R.W, S.W, E.D, R.D, S.D., E.G, R.G and S.G, respectively. In addition, Actinobacteria occupied a large part of the relative abundance in E.G (18.960%) and R.D (22.171%), respectively. In addition, the abundance of Bacteroidetes was high in the R.W, accounting for 19.742%; the abundance of Firmicutes was also high in the R.G, E.G and R.W samples, accounting for 8.735, 7.466 and 6.287%, respectively (Fig. 4a).

In addition, *t*-test analysis of the two groups showed significant differences in the relative abundance of Tenericutes in samples E, G and W (*p* < 0.05) (Fig. 4b). As shown in Fig. 4b, the relative abundance of Tenericutes in E.G was significantly higher than that in E.D, E.W and S.G (*p* < 0.05). The relative abundance of Tenericutes in E.D was significantly higher than that in E.W (*p* < 0.05). Moreover, the relative abundance of Tenericutes in S.W was significantly higher than that in E.W (*p* < 0.05). These findings indicated significant differences in the relative abundance of Tenericutes among plant species and growing year (*p* < 0.05).

**Fig. 5:**
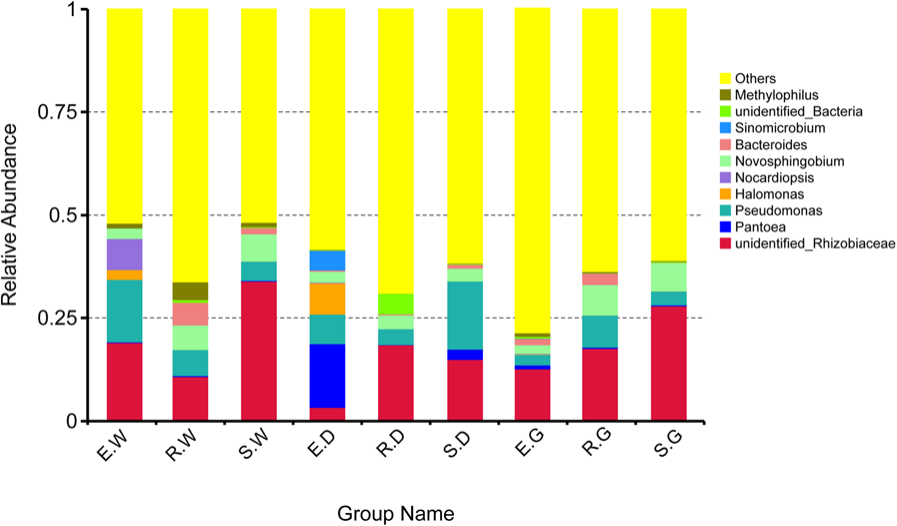
Histograms of relative abundance of the top 10 bacteria at the genera level of taxonomy Description: Ordinate is the relative abundance of bacteria genera; others refers to are sequences with less or not be annotated; abscissa is the group name (E, R and S: years 1, 2, and 3, respectively; W, G and D: *Glycyrrhiza uralensis*, *Glycyrrhiza glabra*, and *Glycyrrhiza inflata*, respectively).

In terms of genus, unidentified-*Rhizobiaceae* was more abundant than other genera (Fig. 5), with the abundance in individual samples ranging from 3.398% (E.D) to 33.985% (S.W). The abundance of *Pseudomonas* was high in the E.W, E.D, R.W, S.D. and R.G samples, accounting for 15.195, 7.165, 6.326, 16.584 and 7.772%, respectively. *Pantoea* (15.386%) and *Halomonas* (7.630%) were found to be the most dominant in E.D sample.

**Fig. 6:**
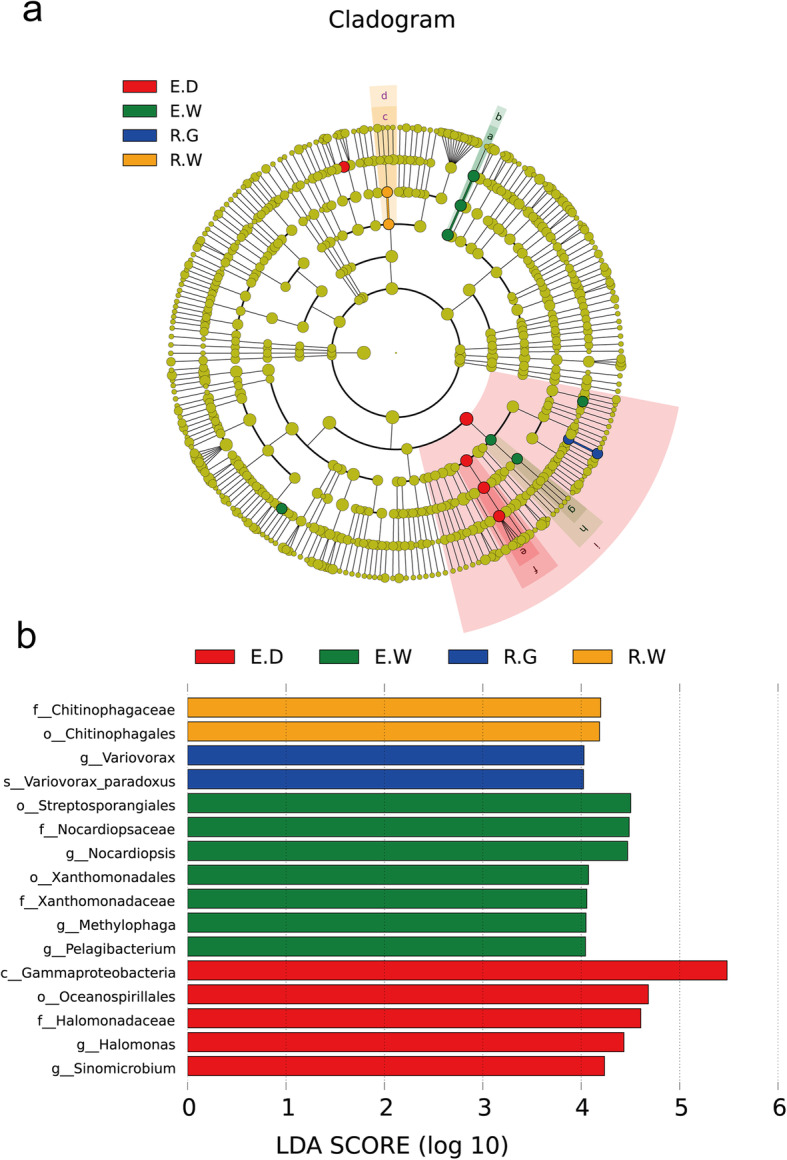
Cladograms (**a**) and LDA value distribution histogram (**b**) in 27 samples. Description: In cladograms (**a**), the circle radiating from inside to outside represents the taxonomic level from the Phylum to the species. Each small circle at a different taxonomic level represents a taxonomic at that level, and the diameter of the small circle is proportionate to the relative abundance of species. The figure shows the species with LDA Score greater than the set value (default setting is 4) (**b**), that is, species with significant differences in different groups. The length of the histogram represents the size of the influence of species with significant differences. The English letters in the figure is the group name (E, R and S: years 1, 2, and 3, respectively; W, G and D: *Glycyrrhiza uralensis*, *Glycyrrhiza glabra*, and *Glycyrrhiza inflata*, respectively).

We used linear discriminant analysis (LDA) effect size (LEfSe) to identify discriminative taxa among different species of medicinal licorices and different growing years. As shown in Fig. 6, the results of LEfSe analysis of all years and species based on the rank sum test revealed a total of 16 biomarkers with significant differences that were contained in the E.D (5 taxa), E.W (7 taxa), R.G (2 taxa) and R.W (2 taxa) groups. No discriminative taxa were observed in year 3. These biomarkers included *Variovorax*, *Nocardiopsis*, *Methylophaga*, *Pelagibacterium*, *Halomonas* and *Sinomicrobium* at the genus taxonomic level (Fig. 6b).

Details of the composition of the top 10 dominant bacteria at other classification levels are listed in Supplementary Table S3. Specifically, Alphaproteobacteria, Gammaproteobacteria, unidentified-Actinobacteria dominate at the class taxonomic level; the dominant species at the order taxonomic level are Rhizobiales, Sphingomonadales and Gammaproteobacteria; the dominant species at the family taxonomic level are Rhizobiaceae, Pseudomonadaceae and Sphingomonadaceae; the dominant species at the species taxonomic level are *Pantoea-brenneri*, *Neorhizobium-huautlense*, and *Pseudomonas-psychrotolerans*.

### Effects of year and species on alpha diversity and beta diversity in the bacterial community of roots

The alpha diversity index of each group is shown in Supplementary Table S4. The results of two-way ANOVA showed that the alpha diversity indexes (Shannon, Simpson, Chao1 and ACE) were not significantly affected by the interaction between plant species and growing year and were not significantly affected by the main effect species and main effect growing year (Table 3).

**Table 3:** Effects of growth years and species on endophytic bacterial community richness index and diversity index

Source		Type III Sum of Squares	Degrees of freedom	Mean Square	F	***p*** value	Partial Eta Squared
year	Shannon	3.157	2	1.578	1.540	0.241	0.146
	Simpson	0.009	2	0.004	1.401	0.272	0.135
	Chao1	21,992.749	2	10,996.375	0.659	0.530	0.068
	ACE	21,955.833	2	10,977.917	0.728	0.496	0.075
species	Shannon	4.650	2	2.325	2.269	0.132	0.201
	Simpson	0.006	2	0.003	0.934	0.411	0.094
	Chao1	41,731.275	2	20,865.638	1.250	0.310	0.122
	ACE	32,540.035	2	16,270.017	1.079	0.361	0.107
year*species	Shannon	7.272	4	1.818	1.774	0.178	0.283
	Simpson	0.016	4	0.004	1.246	0.327	0.217
	Chao1	129,404.800	4	32,351.200	1.938	0.148	0.301
	ACE	121,953.430	4	30,488.357	2.023	0.134	0.310

Description: Description: *P* < 0.05 indicates statistical significance

**Fig. 7:**
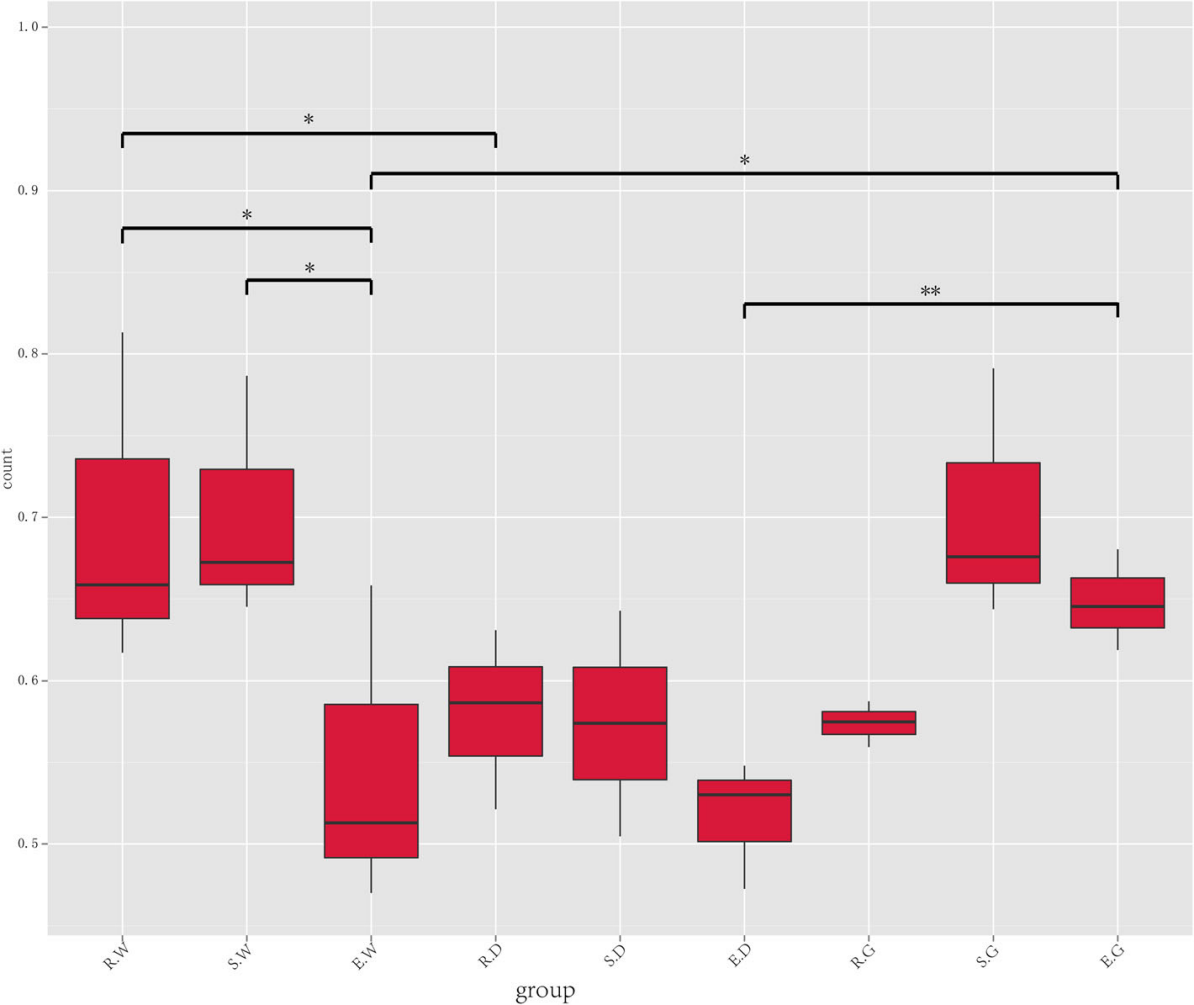
The significance test of the differences of Beta diversity. Description: Ordinate is the Beta diversity; abscissa is the group name (E, R and S: years 1, 2, and 3, respectively; W, G and D: *Glycyrrhiza uralensis*, *Glycyrrhiza glabra*, and *Glycyrrhiza inflata*, respectively). The mark * is significance test *p* < 0.05.

However, beta diversity analysis showed significant differences in the endophytic bacterial community among the different groups. As shown in Fig. 7, Wilcox rank sum test based on the UniFrac distances showed significant differences in beta diversity between E.W and S.W (*P* < 0.05), E.W and R.W (*P* < 0.05), E.W and E.G (*P* < 0.05), R.W and R.D (*P* < 0.05), and E.D and E.G (*P* < 0.01), which indicated the existence of significant differences in endophytic bacterial community of roots of medicinal licorices between different species and different growing years.

### The relationship between the dominant phylum and genus of endophytic bacteria and the bioactive compounds, soil physicochemical properties and leaf nutrition

Spearman correlation analysis showed that there was a significant relationship between dominant bacteria phylum and bioactive compounds, soil physicochemical properties and leaf nutrition (Table 4). Specifically, Proteobacteria showed a very significant negative correlation with PTN; Actinobacteria showed a significant negative correlation with SAN, GIA and LI (*R*^*2*^ < 0; *P* < 0.05); Firmicutes showed a significant positive correlation with PTN and CF; and Acidobacteria showed a significant positive correlation with POC (*R*^*2*^ > 0; *P* < 0.05).

**Table 4:** Spearman correlation analyses testing the relationship between the relative abundance of dominant bacterial phyla in the root of licorice and bioactive compounds, soil physicochemical properties and leaf nutrition

	Proteobacteria	Actinobacteria	Bacteroidetes	Firmicutes	Unidentified-Bacteria	Chlamydiae	Tenericutes	Acidobacteria	Verrucomicrobia	Planctomycetes
SOM	0.098	−0.219	−0.096	−0.190	−0.253	−0.167	−0.305	−0.246	0.092	−0.332
STN	0.242	−0.358	−0.004	−0.178	−0.236	−0.207	− 0.131	−0.252	− 0.020	−0.155
STP	−0.024	−0.236	0.209	0.099	−0.129	0.125	0.165	−0.319	−0.070	− 0.033
STK	−0.220	0.087	0.216	0.091	−0.127	0.060	−0.225	0.111	0.256	−0.116
SNN	0.075	0.154	−0.137	−0.005	− 0.128	−0.241	0.292	0.128	−0.355	−0.026
SAN	0.073	−0.455*	0.250	0.138	0.223	0.107	0.067	−0.068	0.208	−0.222
TS	0.034	0.274	−0.228	0.045	−0.195	−0.276	0.311	0.214	−0.289	0.211
PWC	−0.088	0.111	0.020	0.173	−0.270	−0.094	0.047	0.038	−0.204	−0.046
POC	−0.178	0.016	0.181	0.354	−0.290	−0.225	0.074	0.434*	0.077	0.151
PTN	−0.499**	0.291	0.187	0.430*	0.236	0.008	0.339	0.263	0.066	0.184
PTP	0.022	0.122	−0.134	0.090	−0.162	− 0.177	0.238	0.130	−0.199	0.188
PTK	−0.202	0.218	−0.010	0.267	−0.339	−0.172	0.279	0.117	−0.186	0.214
CF	−0.330	0.206	0.095	0.408*	−0.054	0.170	0.343	0.121	−0.022	0.212
GlA	0.358	−0.463*	0.049	−0.274	0.185	0.040	−0.097	−0.204	− 0.012	−0.100
GTF	0.168	−0.354	0.031	−0.241	0.248	0.083	−0.038	−0.165	0.221	−0.070
LI	0.331	−0.458*	0.079	−0.242	0.189	0.074	−0.111	−0.218	0.037	−0.109

Description: the values are the correlation coefficients. ** means *P* < 0.01; * means *P* < 0.05

**Fig. 8:**
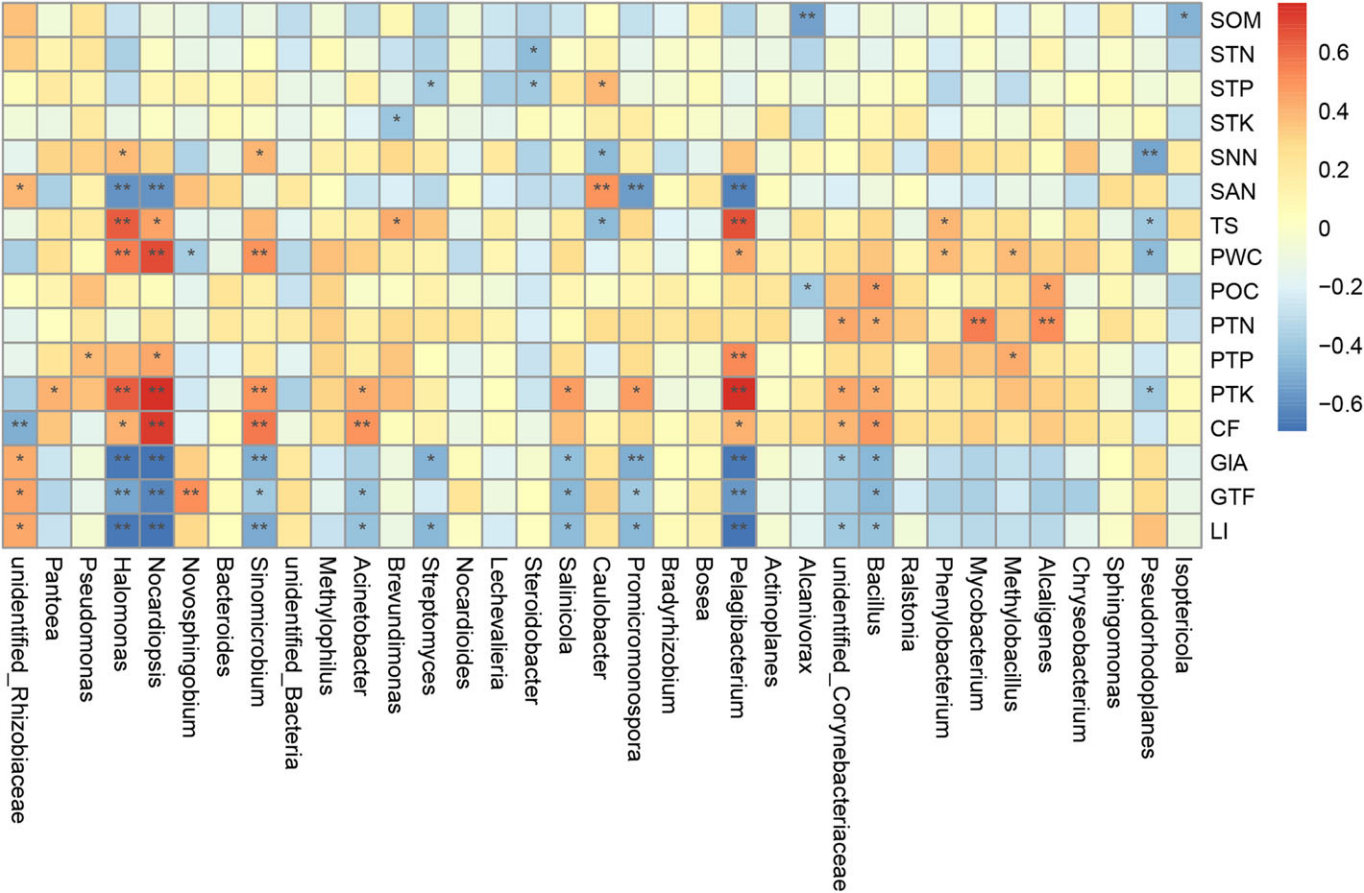
Heat maps of Spearman correlation analysis. Description: Ordinate is the information of environmental factors, and abscissa is the information of species at the genera level of taxonomy. The correlation coefficient r of Spearman is between − 1 and 1, *r* < 0 is negative correlation, *r* > 0 is positive correlation, and the mark * is significance test *p* < 0.05.

As shown in Fig. 8, there was a significant relationship between the dominant bacteria genus and the bioactive compounds of roots, soil physicochemical factors and leaf nutrition. Specifically, unidentified-*Rhizobiaceae* had a significant positive correlation with SAN, GIA, GTF and LI, but a very significant negative correlation with CF; *Pantoea* had a significant positive correlation with PTK; *Pseudomonas* had a significant positive correlation with PTP; *Halomonas* had a significant positive correlation with SNN, TS, PWC, PTK and CF, but a very significant negative correlation with SAN, GIA, GTF and LI; *Nocardiopsis* had a significant positive correlation with TS, PWC, PTP, PTK and CF, but a very significant negative correlation with SAN, GIA, GTF and LI; *Novosphingobium* had a significant negative correlation with PWC, but a very significant positive correlation with GTF; and *Sinomicrobium* had significant positive correlation with SNN, PWC, PTK and CF, but a significant negative correlation with GIA, GTF and LI. It is worth noting that we found that the biomarkers with significant differences at the genus taxonomic level of the dominant bacterial community (including *Nocardiopsis*, *Halomonas* and *Sinomicrobium*) had a significant negative correlation with GIA, GTF, and LI (*R*^*2*^ < 0; *P* < 0.05). Details of the interrelationships between the biomarkers with significant differences in the class, orders, families and genera of dominant bacteria and bioactive compounds, soil physicochemistry and leaf nutrients are shown in Supplementary Table S5. These results showed that the biomarkers with significant differences (except *Methylophaga*) at each classification level were significantly negatively correlated with GIA, GTF, and LI, while all showed a significant positive correlation with PTK.

### Total flavonoids explained the difference in composition and distribution of endophytic bacteria community in the roots of planting licorices to the greatest extent

**Fig. 9:**
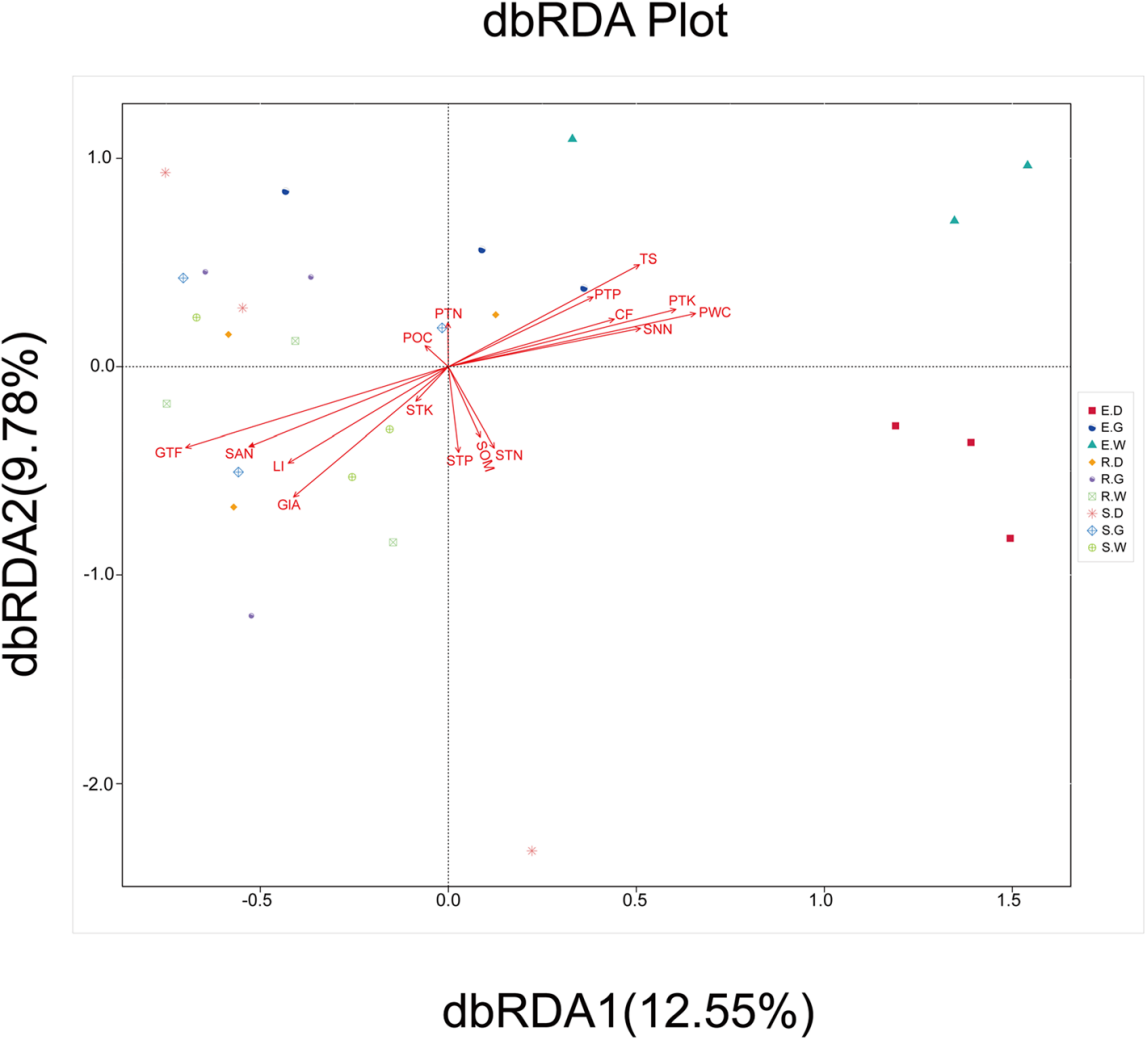
Db-RDA for all groups. Description: Environmental factors are generally represented by arrows. The length of the arrow line represents the degree of correlation between a certain environmental factor and community and species distribution, and the longer the arrow, the greater the correlation. When the angle between the environmental factors is acute, it means that there is a positive correlation between the two environmental factors, while when the angle is obtuse, there is a negative correlation.

Distance-based redundancy analysis (db-RDA) based on the Bray–Curtis distance showed that the bioactive compounds, soil physicochemical and leaf nutrient components had significant effects on the endophytic bacterial community in the roots of medicinal licorices (Fig. 9, Table 5). Specifically, the contents of GIA, GTF, and LI all had a significant effect on the endophytic bacterial community in the roots (*P* < 0.05). Among them, the content of GTF explained the difference in the composition and distribution of endophytic bacterial communities in the roots of cultivated medicinal liquorices to the greatest extent (*r*^*2*^ = 0.638, *P* < 0.01). Among the soil environment factors, TS of soil was identified as the factor that most significantly affects the endophytic bacterial community, followed by SAN and SNN. Among the leaf nutrients factors, PWC was identified as the factor that most significantly affects the endophytic bacterial composition, followed by PTK and PTP. In addition, Mantel tests revealed that the combination of medicinal components is the environmental factor with the greatest correlation with the endophytic bacterial community, indicating that the combination has the greatest impact on the microbial community (*r* = 0.260; *P* = 0.008) (Supplementary Table S6).

**Table 5:** Results for db-RDA testing effects of soil physicochemical properties, leaf nutrients and bioactive compounds on the composition and distribution of bacterial community in licorice root

	***r***^***2***^	***P*** Value
SOM	0.118	0.213
STN	0.163	0.113
STP	0.165	0.110
STK	0.035	0.666
SNN	0.296	0.010
SAN	0.428	0.002
TS	0.497	0.000
PWC	0.498	0.001
POC	0.014	0.849
PTN	0.044	0.607
PTP	0.260	0.028
PTK	0.442	0.001
CF	0.247	0.033
GlA	0.555	0.000
GTF	0.638	0.000
LI	0.393	0.005

Description: *r*^*2*^ is the determinant coefficients of the distribution of the bacterial community by environmental factors

## Discussion

In this study, we explored the effects of planting year (years 1, 2, and 3) and species of medicinal licorices (*Glycyrrhiza uralensis*, *Glycyrrhiza glabra*, and *Glycyrrhiza inflata*) on the accumulation of bioactive compounds. We found that the bioactive compounds were more affected by main effect planting year than main effect species (Table 1 and Fig. 1), which is consistent with the results of Fan et al. [31]. On the one hand, the bioactive compounds of medicinal plants are the products of physiological activities that occur in the process of cell specialization and maturation [26], and their contents increase with the growth and development of plants. On the other hand, most of the active ingredients in medicinal plants are secondary metabolites [32] (including phenolic compounds, total flavonoids and glycyrrhizin), which accumulate during plant growth and development to provide protection against pathogens and neighboring plants [33]. Furthermore, plant growth and development are influenced by climatic conditions (rainfall regimen and temperature). Zoe Bont et al. [34] demonstrated that plants can adapt to changing environments by adjusting the production and maintenance of compounds in their roots. Plants grown for 3 years in this study were subjected to two more years of climate variability than other sample plants, which can stimulate the accumulation of compounds in the roots and accelerate their resistance to environmental pressures. This is also one of reasons why growth year is a factor that influences the accumulation of bioactive compounds in the roots of medicinal plants.

Similarly, the accumulation of bioactive compounds in medicinal licorice roots is easily affected by many factors. In this study, our results showed the components in leaves (PWC, POC, PTN, PTP, PTK and CF) were negatively correlated with the contents of glycyrrhizic acid (GIA), liquiritin (LI) and total flavonoids (GTF) in roots (Table 2 and Fig. 2). Thus, these findings indicated that resource allocation and nutrient competition occur among roots and leaves of medicinal licorices. At the same time, we showed that the content of glycyrrhizic acid (GIA), liquiritin (LI) and total flavonoids (GTF) are negatively correlated with TS in soil (Table 2). We speculate that this effect is mainly because the high concentration of salts (mostly ions like Na^+^) in the soil can decrease the uptake of water and nutrients by plants, as well as the rate of photosynthesis [35], resulting in ion and osmotic stress [36], thus inhibited the accumulation of bioactive compounds. However, appropriate salt stress can increase the accumulation of bioactive compounds in licorice by upregulating proteins involved in the related biosynthesis pathways to stimulate the production of glycyrrhizic acid, liquiritin and total flavonoids and other bioactive compounds [37, 38]. Wan [39] and Yang [40] et al. demonstrated that appropriate salt stress stimulated the glycometabolism of *Glycyrrhiza*, accelerated the decomposition of substances, and promoted the formation and accumulation of glycyrrhizic acid, liquiritin and total flavonoids in medicinal licorices, while high concentration and long-term salt stress inhibited the production of bioactive compounds.

In addition, our results also showed that GIA, LI and GTF had a significant positive correlation with SAN, but have no significant correlation with SNN (Table 2). We speculate that this is related to the concentration of ammonium nitrogen and nitrate nitrogen in soil and the efficiency of root absorption [41]. In addition, Pei et al. [42] reported the highest content of total flavonoids (GTF) in the root of licorices when SNN: SAN was 0/100, which was significantly higher than that the contents when SNN: SAN was 100/0 and 50/50. These findings showed that different forms and ratios of nitrogen can affect the quality of *Glycyrrhiza*, indicating that the nitrogen sources in the soil in the form of ammonium nitrogen and nitrate nitrogen contribute collectively to the accumulation of bioactive compounds in medicinal licorices. On the other hand, nitrogen fixation is a result of the symbiotic relationship between rhizobia and host plants, which can facilitate the absorption of nitrogen from the air and soil by host plants. Some studies [43, 44] showed that rhizobia form nodules in the root of *glycyrrhiza* by symbiosis and facilitate nitrogen fixation by the hosts in exchange for nutrients. The amount of nitrogen fixation by rhizobia is affected by the host’s demand and yield potential for nitrogen, the compatibility of both symbiotic partners and the availability of nitrogen in the soil. Therefore, clarifying the role of soil factors in the growth of medicinal licorices will provide a theoretical basis for the synthesis of bioactive compounds and rational utilization of medicinal plants in production practice.

In this study, we investigated the composition and diversity of endophytic bacterial communities in three medicinal licorices roots using high-throughput sequencing technology, which provides a large amount of data with more accuracy than that obtained in previous studies using traditional technology [45, 46]. Our study showed no significant differences in the alpha diversity index, which is used to describe the diversity and complexity of the bacterial community, between the interaction effect planting year and species of medicinal licorices (Table 3). However, our results showed the indexes of endophytic bacterial diversity in year 3 were slightly lower than those in year 2 (Supplementary Table S4), which, combined with the observation that growth years had a significant impact on the contents of GIA, GTF and LI, and showed an upward trend with the increasing number of growing year (Fig. 1). We speculate that the accumulation of bioactive compounds in roots regulates the endophytic bacterial diversity and that this is mediated via mechanisms that reduce the relative abundance through the antibacterial effects of bioactive compounds or chemical signals that negatively affect growth [10]. Although the mechanism by which bioactive compounds regulate endophytic bacteria is still unclear, this discovery may form the basis of further in-depth research. In addition, studies have shown that plant organs, rainfall and temperature are the main drivers of endophytic components [47]. In this study, climatic conditions, especially rainfall, in year 2 were significantly higher than those in year 3 (Supplementary Table S1). This difference may change endophytic bacteria diversity, suggesting that these species are likely to be affected by climate changes in the near future.

Our results showed specific microbiomes in 27 samples of medicinal licorices. Proteobacteria was the dominant phylum in all samples, followed by Actinobacteria and Bacteroidetes (Fig. 4a), which is consistent with the results of Cheng et al. [48] The phylum Proteobacteria, which constitutes the largest and phenotypically most diverse phylogenetic lineage at present [49], is an important group of microorganisms in evolutionary, geological, and environmental terms [50]. Numerous studies have confirmed that Proteobacteria is the main dominant phylum of the endophytic bacterial communities associated with plants ranging from economic crops, such as soybean [51] and tomato [52], to ornamental plants, such as peony [53].

Among the 27 samples of medicinal licorices analyzed in this study, unidentified *Rhizobiaceae* was the dominant genus (Fig. 5). Unidentified*-Rhizobiaceae*, which may be non-culturable or not studied, may be a bacterium of potential importance in the endophytic bacterial community of licorice, because of the nitrogen fixation function of rhizobia, which has become a research hotspot in legumes [54]. Wei et al. [55] demonstrated high tolerance to NaCl, pH and temperature in a large number of isolated rhizobia from *Glycyrrhiza uralensis* Liu et al. [49] showed that inoculation with rhizobia improved the growth of *Glycyrrhiza uralensis* and increased the dry weight of the underground rhizome. These studies all showed that beneficial rhizobia coexist with and promote the growth of *Glycyrrhiza* medicinal licorices. In addition, our results also showed a significant positive correlation between unidentified-*Rhizobiaceae* and the content of GIA, GTF and LI (Fig. 8). Although the scope of beneficial and culturable *Rhizobiae* is still unclear at present, this information provides a direction for future research on improving the efficacy of medicinal licorices.

Although many studies the composition of plant endophytic bacterial communities are based on Illumina MiSeq technology [56], few studies have focused on the influence of planting year and species on the composition of endophytic bacterial communities in *Glycyrrhiza*. Our results show that the endophytic bacteria community is affected by the planting year and species of *Glycyrrhiza* (Fig. 7), which helps to fill the knowledge gap in this field. Endophytic bacteria exist in plant species as part of the root microbiome. The endophytic bacteria community (species diversity: abundance and relative abundance) in plants is dynamic and affected by abiotic and biological factors, such as soil conditions, biogeography and plant species [57]. In this study, the content of bioactive compounds in roots was shown to be the driving factor in differences in the composition and distribution of endophytic bacteria community (Fig. 9, Table 5, Supplementary Table S6), with the total flavonoid content shown to explain the differences in the composition and distribution of endophytic bacteria community to the greatest extent. Flavonoids, which are a variety of polyphenolic compounds produced in plants by secondary metabolism, can act as a signal of interaction between plants and many microorganisms [58, 59]. For example, flavonoids can act as chemoattractants and inducers of nodulation (nod) and other genes in rhizobia [60]. In recent years, a large number of studies including research by Márton Szoboszlay et al. [61] have also shown that flavonoids have an impact on bacterial communities.

The endophytic bacterial composition differed among the samples, and our study also indicates that the endophytic bacterial community in the roots of medicinal licorices is determined by the bioactive compounds in the roots. In addition, this result is consistent with the hypothesis that the physical and chemical factors in the soil and leaf nutrition dictate the composition of the endophytic bacterial population, although further studies are required to characterize the roles of these endophytic bacteria. Our study provides useful information for the development of strategies to improve the production and quality of medicinal licorices.

## Conclusions

In this study, numerous endophytic bacteria communities were detected in the roots of medicinal licorices based on high-throughput sequencing. Furthermore, we identified significant differences in the relative abundance of some endophytic bacteria among plant species and growing year. Furthermore, the contents of glycyrrhizic acid, liquiritin and total flavonoids in the roots of medicinal licorices increased significantly with the main effect planting year, and the total flavonoid content shown to explain the differences in the composition and distribution of endophytic bacteria community to the greatest extent.

## Methods

### 4.1 Sample collection

In May 2016, the seeds of three species of medicinal licorices (*Glycyrrhiza uralensis*, *Glycyrrhiza inflata*, and *Glycyrrhiza glabra*) were purchased from Xinjiang Beiling licorice Technology Co., Ltd. (Xinjiang, China), and were sown in the south of Yanqi County (86°17′33″ E, 42°11′34″ N), Xinjiang, China. There were nine separate sample plots (4 × 4 m per plot, 3 plots × 3 replicates for each species of licorices). The fields were ploughed each year before sowing and each sample plot was harrowed to 20 cm. The licorice plant seeds were watered immediately after sowing, in the middle growth period and at the stage of maturity. Seeds were sown in this way every year for 3 years. Details of the temperature, rainfall and soil factors during the experimental period are shown in Supplementary Table S1. Field sampling was conducted in August 2017, August 2018 and August 2019, when liquorice roots and rhizosphere soil (depth 0–40 cm) were collected. To ensure that the experiment was representative, three well-grown licorice roots were randomly selected and cut out with sterile scissors. Each root was divided into two parts: one part was placed into a ziplocked bag for the determination of the bioactive compounds in the root, while the other part was placed into a sterile bag and quickly transported on a piece of ice to the laboratory. To eliminate the interference of other microorganisms, the surface of roots was sterilized in the laboratory by first rinsing soil from the roots under running water followed by washing with sterile distilled water. The roots were then soaked in 75% alcohol for 30 s for surface disinfection, then washed five times with sterile distilled water before soaking in 5% sodium hypochlorite for 5 min. Finally the roots were washed five times with sterile distilled water and air-dried under sterile conditions. To confirm that the surface sterilization process was successful, the last rinse solution was inoculated onto a Luria–Bertani (LB) medium and cultured at 28 °C for 72 h. No bacterial growth confirmed that the surface sterilization was successful. All root samples were immediately placed on ice and then stored at liquid nitrogen prior to total DNA extraction. Soil samples from the rhizosphere were air-dried and sieved through a 2-mm mesh. The following soil physicochemical characteristics were analyzed according to the methods described by the Bao et al. [62]: The content of organic matter (SOM) was determined by external heating with potassium dichromate. The total nitrogen (STN) content was determined using the perchloric acid-sulfuric acid digestion method. The total phosphorus (STP) content was determined by acid digestion (molybdenum-antimony colorimetry). The total potassium (STK) content was determined by acid digestion (atomic absorption spectrometry). The total salt (TS) content was determined by atomic absorption spectrometry. Nitrate nitrogen (SNN) and ammonium nitrogen (SAN) contents were analyzed using 0.01 M calcium chloride extraction. In addition, leaf samples, were air-dried to constant weight, ground to a powder using a pestle and mortar and passed through a 60-mesh sieve. The following plant nutritional components were then analyzed: The content of organic carbon (POC) was determined by external heating with potassium dichromate. Water content (PWC) was determined by weighing. The total nitrogen (PTN) content was determined using the perchloric acid-sulfuric acid digestion method. The total phosphorus (PTP) content was determined by acid digestion (molybdenum-antimony colorimetry method). The total potassium (PTK) content was determined by acid digestion (atomic absorption spectrometry). The crude fiber (CF) content was determined using the acid-base detergent method. All the samples were labeled by combination with letters and numbers, with the first letter representing the age of the medicinal licorices (E, R and S: years 1, 2, and 3, respectively), the second letter representing the species (W, G and D: *Glycyrrhiza uralensis*, *Glycyrrhiza glabra*, and *Glycyrrhiza inflata*, respectively), and the third number representing the replicate number. For example, E.W.3 represents the third repetition of *Glycyrrhiza uralensis* in the first year.

### Determination of active components

The root samples were dried at 60 °C for 72 h to constant weight (it has been confirmed that glycyrrhizic acid (GIA) and liquiritin (LI) do not decompose at this temperature [63]). The dried root samples were ground to a powder with a pestle and mortar and passed through a 60 mesh sieve. An aliquot (0.2 g) of powdered root sample was extracted with 71% chromatographic methanol in an ultrasonic bath (250 W, 40 kHz) at room temperature [64]. The extract was then centrifuged at 12,000 rpm for 10 min and the supernatant was filtered (0.22-μm pore size) (Agilent, USA). The GIA and LI contents in the dried root samples (0.2 g) of the medicinal licorices were determined by high-performance liquid chromatography (HPLC, Agilent-1260 Infinity, USA) using an Agilent ZORBAX SB-C18 column (150 mm × 4.6 mm, 5 μm) with mobile phase (chromatographic methanol: ultra-pure water: 36% glacial acetic acid = 71:28:1) and mobile phase (acetonitrile:0.5% glacial acetic acid = 1:4) respectively, and a gradient elution flow rate of 1.0 mL•min-1. GIA and LI were detected at 254 nm and 276 nm, respectively. The injection volume was 5 μL and the column temperature was 30 °C. The GIA and LI reference materials (CAS#1405-86-3 and CAS#551–15-5, respectively) were purchased from Solarbio and used for calibration purposes. The total flavonoid content (GTF) in medicinal licorices was determined by ultraviolet spectrophotometry at 334 nm with the LI standard (CAS#551–15-5) as the control.

### DNA extraction and library construction

After immersion in liquid nitrogen, genomic DNA was extracted from the samples using the DNA Quick Plant System kit (Tiangen, China) according to the manufacturer’s instructions. The purity and concentration of DNA were evaluated using a NanoDrop2000 (Thermo Fisher Scientific, USA). According to the concentration, each DNA sample was diluted a final concentration to 1 ng/μL with sterile distilled water for use as a DNA template. The 16S rDNA genes of the V4 region were amplified using specific primers (515F: 5′-GTGCCAGCMGCCGCGGTAA-3′ and 806R: 5′-GGACTACHVGGGTWTCTAAT-3′) with barcodes [65]. PCR analyses were carried out with Phusion® High-Fidelity PCR Master Mix and GC Buffer (New England Biolabs) to ensure amplification efficiency and accuracy. PCR runs started at at 95 °C for 3 min, followed by 30 cycles of 95 °C for 30 s, 55 °C for 30 s, 72 °C for 30 s and a final extension step at 72 °C for 5 min.

The PCR product was mixed with the same volume of 1× loading buffer (containing SYBR green) and then was detected by 2% agarose gel electrophoresis. The PCR product was purified from the target strip using a Gel Extraction Kit (Qiagen, Germany). The libraries were constructed using a TruSeq®DNA PCR-Free Sample Preparation Kit (Illumina, USA) according to the manufacturer’s instructions, and index codes were added. The library quality was assessed on the Qubit® 2.0 Fluorometer (Thermo Scientific) and Agilent Bioanalyzer 2100 system. Finally, Amplicon sequencing was performed using the Illumina HiSeq2500 platforms at the Beijing Compass Biotechnology Co., Ltd. (Beijing, China).

### Bioinformatics analysis

Paired-end reads were assigned to samples based on their unique barcode and truncated by cutting off the barcode and primer sequence. The paired-end reads of each sample were spliced using FLASH (V1.2.7) [66] and then assembled to generate raw tags. To avoid the influence of non-microbiota sequences (such as, chloroplast and mitochondrial sequences), the raw sequences were further filtered by QIIME (V1.9.1) [67] to remove non-microbiota taxa before subsequent analysis. Then raw tags were subjected to a strict quality filtering process using QIIME to obtain high-quality clean tags [68]. Effective tags were obtained by comparison of the clean tags sequence with the UCHIME Algorithm [69] and Gold database to detect and remove chimeric sequences.

UPARSE software (UPARSE v7.0.1001) was used to cluster the effective tags of all samples into the same operational taxonomic units (OTUs) with ≥97% identity, and taking the sequence with the highest frequency as the representative sequence of each OTU. The taxonomic information for each representative sequence was annotated using the SILVA database (threshold value set to 0.8–1), and multiple sequence alignment was performed using MUSCLE (Version 3.8.31) software to study the phylogenetic relationship of the representative sequences of OTUs among the 27 root samples. OTU abundance information was normalized using a standard sequence number corresponding to the sample with the lowest sequences (49,096 reads for sample E.W.1). Subsequent analysis of alpha diversity and beta diversity were performed based on this output normalized data. The raw sequence reads have been deposited in the Sequence Read Archive (SRA) database of NCBI (SRR12483393 to SRR12483419)(https://dataview.ncbi.nlm.nih.gov/object/PRJNA658331?reviewer=pi4poruikv7bnsshn4kaq4n3tc).

### Statistical analysis

The average of the three replicate samples was used to generate the histograms of relative abundance (phylum and genus). R software (Version 2.15.3) was used for alpha diversity index and beta diversity analyses, rarefaction curve generation, Spearman correlation analysis of heat maps, Mantel tests and distance-based redundancy analysis (db-RDA). LEfSe software was used for linear discriminant analysis (LDA) and effect size (LEfSe) analysis with the default filtering value of LDA score set at 4. Statistical analysis (including one-way analysis of variance (ANOVA) and Spearman correlation analysis) were performed with SPSS 19.0 (IBM Inc., Armonk, USA), two-way ANOVA was used to analyze the effects of different year and licorice species on the contents of bioactive compounds. Spearman correlations (r) were run among the bioactive compounds, the soil physicochemical properties and nutritional components of leaves.

## Supplementary information

**Additional file 1: Table S1.** Soil and plant factors of samples. Group name (E, R and S: years 1, 2, and 3, respectively; W, G and D: *Glycyrrhiza uralensis*, *Glycyrrhiza glabra*, and *Glycyrrhiza inflata,* respectively). **Table S2.** Sequencing results of each sample. Raw reads refers to the sequence filtering out low-quality bases; clean reads refers to the sequence finally used for subsequent analysis after filtering chimeras; base refers to the number of bases of final clean reads; Avglen refers to the average length of clean reads. Q20 refers to the percentage of bases whose quality value is greater than 20 (sequencing error rate is less than 1%); GC (%) refers to the content of GC bases in clean reads; effective (%) refers to the percentage of the number of clean reads and the number of raw reads. Sample name: E, R and S: years 1, 2, and 3, respectively; W, G and D: *Glycyrrhiza uralensis*, *Glycyrrhiza glabra* and *Glycyrrhiza inflata*, respectively; the third number representing the replicate number. **Table S3.** Composition information of dominant bacteria at each classification level. Others: The sum of the undefined and unannotated parts. Group name: E, R and S: years 1, 2, and 3, respectively; W, G and D: *Glycyrrhiza uralensis*, *Glycyrrhiza glabra* and *Glycyrrhiza inflata*, respectively; the third number representing the replicate number. **Table S4.** The alpha diversity indices in each group. Community richness was identified using the Chao1 and ACE estimator. Community diversity was identified using the Shannon and Simpson indexes. Sequencing depth was characterized by Good’s coverage, good’s coverage estimator values ranged from 99.6 to 99.8%, indicating that the sequence numbers per group were high enough. Group: E, R and S: years 1, 2, and 3, respectively; W, G and D: *Glycyrrhiza uralensis*, *Glycyrrhiza glabra* and *Glycyrrhiza inflata*, respectively. **Table S5.** Spearman correlation analyses testing the relationship between the Biomarker and the relationship between the physicochemical properties of soil, leaf nutrients and bioactive compounds of licorice root. The values are the correlation coefficients. ** means *P* < 0.01; * means *P* < 0.05. **Table S6.** Correlations between environmental factors and endophytic bacterial communities in each group. Variable is the information of environmental factors, *r* is the correlation coefficient, and *P* value is the *p*-value of significance test. The larger *r* value is, the greater the correlation between environmental factors and species abundance information is. *P* < 0.05 indicates statistical significance.

## Data Availability

All data generated or analysed during this study are included in this published article and its supplementary information files.

## Abbreviations

HPLC: High-performance liquid chromatography
LB: Luria-Bertani
GIA: Glycyrrhizic acid
LI: Liquiritin
GTF: Total flavonoid
SOM: Organic matter
STN: Total nitrogen
STP: Total phosphorus
STK: Total potassium
TS: Total salt
SNN: Nitrate nitrogen
SAN: Ammonium nitrogen
POC: Organic carbon
PWC: Water content
PTN: Total nitrogen
PTP: Total phosphorus
PTK: Total potassium
CF: Crude fiber
OTUs: Operational taxonomic units
db-RDA: Distance-based redundancy analysis
LDA: Linear discriminant analysis
LEfSe: Linear discriminant analysis effect size
ANOVA: Analysis of variance

## References

[1] Harrison JG, Griffin EA (2020). The diversity and distribution of endophytes across biomes, plant phylogeny and host tissues: how far have we come and where do we go from here?. Environ Microbiol.

[2] Hardoim PR, Van Overbeek LS, Berg G (2015). Pirttil? AM, Compant S, Campisano a, D?Ring M, Sessitsch a. the hidden world within plants. Ecological and evolutionary considerations for defining functioning of microbial Endophytes. Microbiol Mol Biol Rev.

[3] Ryan RP, Kieran G, Ashley F, Ryan DJ, Dowling DN (2008). Bacterial endophytes: recent developments and applications. FEMS Microbiol Lett.

[4] Schulz BJE, Boyle C, Christine JC, Sieber TN (2006). Microbial root endophytes.

[5] Walia A, Guleria S, Chauhan A, Mehta P (2017). Endophytic Bacteria.

[6] Wani ZA, Ashraf N, Mohiuddin T, Riyaz-Ul-Hassan S (2015). Plant-endophyte symbiosis, an ecological perspective. Appl Microbiol Biotechnol.

[7] Jasim B, Jimtha CJ, Jyothis M, Radhakrishnan EK (2013). Plant growth promoting potential of endophytic bacteria isolated from Piper nigrum. Plant Growth Regul.

[8] Lata C, Gond SK, White JF (2018). Induction of abiotic stress tolerance in plants by endophytic microbes. Lett Appl Microbiol.

[9] Yaish MW, Antony I, Glick BR (2015). Isolation and characterization of endophytic plant growth-promoting bacteria from date palm tree (*Phoenix dactylifera* L.) and their potential role in salinity tolerance. Antonie Van Leeuwenhoek.

[10] Bibi F, Yasir M, Song GC, Lee SY, Chung YR (2012). Diversity and characterization of Endophytic Bacteria associated with tidal flat plants and their antagonistic effects on Oomycetous plant pathogens. Plant Pathol J.

[11] Lodewyckx C, Vangronsveld J, Porteous F, Moore ERB, Taghavi S, Mezgeay M, Der Lelie DV (2002). Endophytic bacteria and their potential applications. Crit Rev Plant Sci.

[12] Khan S, Afzal M, Iqbal S, Khan QM (2013). Plant–bacteria partnerships for the remediation of hydrocarbon contaminated soils. Chemosphere..

[13] Senthilkumar M, Anandham R, Madhaiyan M, Venkateswaran V, Sa T (2011). Endophytic Bacteria: Perspectives and Applications in Agricultural Crop Production.

[14] Chebotar VK, Shcherbakov AV, Maslennikova SN, Zaplatkin AN, Kanarskiy AV, Zavalin AA (2016). Endophytic bacteria of woody plants as the basis of complex microbial preparations for agriculture and forestry. Russ Agric Sci.

[15] Berg G (2009). Plant–microbe interactions promoting plant growth and health: perspectives for controlled use of microorganisms in agriculture. Appl Microbiol Biotechnol.

[16] Bartels D, Sunkar R (2005). Drought and salt tolerance in plants. Crit Rev Plant Sci.

[17] Amann RI, Ludwig W, Schleifer KH (1995). Phylogenetic identification and in situ detection of individual microbial cells without cultivation. Microbiol Rev.

[18] Monot M, Orgeur M, Camiade E, Brehier C, Dupuy B (2014). COV2HTML: a visualization and analysis tool of bacterial next generation sequencing (NGS) data for postgenomics life scientists. Omics A J Integr Biol.

[19] Akinsanya MA, Goh JK, Lim SP, Ting ASY (2015). Metagenomics study of endophytic bacteria in Aloe vera using next-generation technology. Genomics Data.

[20] Tao W, Duan J, Zhao R, Li X, Yan H, Li J, Guo S, Yang N, Tang Y (2013). Comparison of three officinal Chinese pharmacopoeia species of Glycyrrhiza based on separation and quantification of triterpene saponins and chemometrics analysis. Food Chem.

[21] Asl MN, Hosseinzadeh H (2010). Review of pharmacological effects of Glycyrrhiza sp. and its bioactive compounds. Phytother Res.

[22] Xueyan G, Wenquan W, Shengli W, Weidong LI (2009). Review of pharmacological effects of Glycyrrhiza radix and its bioactive compounds. Chin J Chin Mater Med.

[23] Baltina LA (2003). Chemical modification of glycyrrhizic acid as a route to new bioactive compounds for medicine. Curr Med Chem.

[24] Honda S, Tominaga Y, Yokota S. Licorice Flavonoids. USA: Wiley-Blackwell; 2009.

[25] Aoki F, Honda S, Kishida H, Kitano M, Arai N, Tanaka H, Yokota S, Nakagawa K, Asakura T, Nakai Y (2007). Suppression by licorice flavonoids of abdominal fat accumulation and body weight gain in high-fat diet-induced obese C57BL/6J mice. Biosci Biotechnol Biochem.

[26] Li Y, Wu H (2018). The research Progress of the correlation between growth development and dynamic accumulation of the effective components in medicinal plants. Chinese Bull Bot.

[27] Da-Cheng H, Pei-Gen X (2015). Genomics and Evolution in Traditional Medicinal Plants: Road to a Healthier Life. Evol Bioinformatics Online.

[28] Yin S, Zhang Y, Gao W, Wang J, Liu H (2014). Effects of nitrogen source and phosphate concentration on biomass and metabolites accumulation in adventitious root culture of Glycyrrhiza uralensis Fisch. Acta Physiol Plant.

[29] Sun ZR, Wang WQ, Ma CH (2004). The underground part growth distribution pattern of Glycyrrhiza uralensis and its effects on glycyrrhizinic acid content. Chin J Chin Mater Med.

[30] Wen-Zhi AN, Ling-Min Z, Jian-Jun X, Xi-Yu Z, Fa-Yuan Z, Yong-Gang L. Study on the root distribution pattern and biomass of the cultivated Glycyrrhiza uralensis. Pratacultural Ence. 2007;7:51–4.

[31] Ming F, Ainong C, Xiaojun J, Leijie J, Han Z (2016). Growth and accumulation of active components of licorice in different growth years in semiarid regions of middle of Gansu Province. Acta Agriculturae Boreali-occidentalis Sinica.

[32] Efferth T, Koch E (2011). Complex interactions between phytochemicals. The multi-target therapeutic concept of Phytotherapy. Curr Drug Targets.

[33] Demain AL, Fang A (2000). The natural functions of secondary metabolites. Adv Biochem Eng Biotechnol.

[34] Bont Z, Züst T, CCM A, Huber M, Erb M. Heritable variation in root secondary metabolites is associated with recent climate. J Ecol. 2020. 10.1111/1365-2745.13441.

[35] Verma N, Shukla S (2015). Impact of various factors responsible for fluctuation in plant secondary metabolites. J Appl Res Med Aromatic Plants.

[36] Sakamoto M, Suzuki T (2015). Effect of Root-Zone Temperature on Growth and Quality of Hydroponically Grown Red Leaf Lettuce (*Lactuca sativa* L. cv. Red Wave). Am J Plant Ences.

[37] Zahra S, Ali A, Masoud T, Sohani MM (2019). Triterpenoid gene expression and phytochemical content in Iranian licorice under salinity stress. Protoplasma.

[38] Wang C, Chen L, Cai Z, Chen C, Mei Y (2020). Comparative proteomic analysis reveals the molecular mechanisms underlying the accumulation difference of bioactive constituents in Glycyrrhiza uralensis Fisch under salt stress. J Agric Food Chem.

[39] Chun-Yang W, Dan W, Jun-Ling H, Wen-Quan W, Wei-Dong LI (2011). Effects of NaCl Stress on Growth,Physiological Index and Content of Effective Composition of Glycyrrhiza uralensis. Chin J Exp Tradit Med Formulae.

[40] Xiu-Hong Y, Jian-Min LI, Xue-Hui D, Liu-Sheng D. Effects of Exogenous Glycyrrhizinic Acid on the Seedling Growth,Glycyrrhizinic Acid Content of Roots and Some Physiological Indexes of Glycyrrhiza uralensis Fisch. Seedling under NaCl Stress. Plant Physiol Commun. 2006;3:441–4.

[41] Anthony D (2003). M., glass. Nitrogen use efficiency of crop plants: physiological constraints upon nitrogen absorption. Crit Rev Plant Sci.

[42] Wenmei P, Senjun Z, Jing W. Effect of different nitrogen forms and ratios on yield and quality of licorice. Chin Agric Ence Bull. 2011;28:184–7.

[43] Cai T, Cai W, Zhang J, Zheng H, Zhu J (2010). Host legume-exuded antimetabolites optimize the symbiotic rhizosphere. Mol Microbiol.

[44] Li L, Hanna S, Leone M, Gehong W, Kristina L, RL A (2012). Biogeography of symbiotic and other endophytic bacteria isolated from medicinal Glycyrrhiza species in China. FEMS Microbiol Ecol.

[45] Mohamad OAA, Li L, Jin-Biao M, Shaimaa H, Lin X, Jian-Wei G, Rasulov BA, Yong-Hong L, Hedlund BP, Wen-Jun L (2018). Evaluation of the Antimicrobial Activity of Endophytic Bacterial Populations From Chinese Traditional Medicinal Plant Licorice and Characterization of the Bioactive Secondary Metabolites Produced by Bacillus atrophaeus Against Verticillium dahliae. Front Microbiol.

[46] Xiao-Li R. Isolation of Endophytic Bacteria from Glycyrrhiza and identifying of antagonistic Bacteria. Microbiology. 2007;4:700–4.

[47] Gomes T, Pereira JA, Benhadi J, Lino-Neto T, Baptista P (2018). Endophytic and epiphytic Phyllosphere fungal communities are shaped by different environmental factors in a Mediterranean ecosystem. Microb Ecol.

[48] Cheng X, Li W, Wang Y, Yang H, Lou K (2009). Endophytic bacterial diversity in Glycyrrhiza inflata bat. From Xinjiang by culture-independent method. Acta Microbiol Sin.

[49] Qian L, Shi-Hao Z, Lai-Fu W, Wen-Xin C (2005). Study on screening of high efficient strains of rhizobium on Glycyrrhiza. J Heb Acad Ences.

[50] Marín I (2015). Proteobacteria.

[51] Okubo T, Ikeda S, Kaneko T, Eda S, Mitsui H, Sato S, Tabata S, Minamisawa K (2009). Nodulation-dependent communities of culturable bacterial endophytes from stems of field-grown soybeans. Microbes Environ.

[52] Romero FM, María M, Pieckenstain FL (2014). The communities of tomato (Solanum lycopersicum L.) leaf endophytic bacteria, analyzed by 16S-ribosomal RNA gene pyrosequencing. FEMS Microbiol Lett.

[53] Ruixian Y, Ping L, Wenyu Y (2017). Illumina-based analysis of endophytic bacterial diversity of tree peony (Paeonia sect. Moutan) roots and leaves. Braz J Microbiol.

[54] Willems A (2006). The taxonomy of rhizobia: an overview. Plant Soil.

[55] Wei GH, Yang XY, Zhang ZX, Yang YZ, Lindsroem K (2008). Strain Mesorhizobium sp. CCNWGX035: a stress-tolerant isolate from Glycyrrhiza glabra displaying a wide host range of nodulation. Pedosphere..

[56] Compant S, Clément C, Sessitsch A (2009). Plant growth-promoting bacteria in the rhizo- and endosphere of plants: their role, colonization, mechanisms involved and prospects for utilization. Soil Biol Biochem.

[57] Gaiero JR, Mccall CA, Thompson KA, Day NJ, Best AS, Dunfield KE (2013). Inside the root microbiome: bacterial root endophytes and plant growth promotion. Am J Bot.

[58] Shaw LJ, Morris P, Hooker JE (2010). Perception and modification of plant flavonoid signals by rhizosphere microorganisms. Environ Microbiol.

[59] Mathesius HU (2012). The role of flavonoids in root-rhizosphere signalling: opportunities and challenges for improving plant-microbe interactions. J Exp Bot.

[60] Cooper JE (2004). Multiple responses of rhizobia to flavonoids during legume root infection. Adv Bot Res.

[61] Márton S, Alison W-M, Luke M (2016). The Effect of Root Exudate 7,4′-Dihydroxyflavone and Naringenin on Soil Bacterial Community Structure. PLoS One.

[62] Bao SD. Soil Agro-chemistrical Analysis. China: China Agriculture Press; 2008. p. 22–196.

[63] Jing Q, Zuliang L, Yanpeng L, Guangxi R, Chunsheng L, Xiaojun M (2017). Effect of Abscisic acid on accumulation of five active components in root of Glycyrrhiza uralensis. Molecules..

[64] Xie W, Hao Z, Yu M, Wu Z, Zhao A, Li J, Zhang X, Chen B (2019). Improved phosphorus nutrition by arbuscular mycorrhizal symbiosis as a key factor facilitating glycyrrhizin and liquiritin accumulation in Glycyrrhiza uralensis. Plant Soil.

[65] Berry D, Ben Mahfoudh K, Wagner M, Loy A (2012). Barcoded primers used in multiplex amplicon pyrosequencing Bias amplification. Appl Environ Microbiol.

[66] Tanja M, Steven S (2011). FLASH: fast length adjustment of short reads to improve genome assemblies. Bioinformatics.

[67] Caporaso JG, Kuczynski J, Stombaugh J, Bittinger K, Bushman FD, Costello EK, Fierer N, Peña AG, Goodrich JK, Gordon JI (2010). QIIME allows analysis of high-throughput community sequencing data. Nat Methods.

[68] Bokulich NA, Subramanian S, Faith JJ, Gevers D, Gordon JI, Knight R, Mills DA, Caporaso JG (2013). Quality-filtering vastly improves diversity estimates from Illumina amplicon sequencing. Nat Methods.

[69] Robert E, Brian H, Jose Clemente C (2011). UCHIME improves sensitivity and speed of chimera detection. Bioinformatics.

