# Supplementary material for: Root-associated endophytic bacterial community composition and structure of three medicinal licorices and their changes with the growing year
Source: BMC Microbiol. 2020 Sep 21;20:291. doi: 10.1186/s12866-020-01977-3 (PMC7507641; doi:10.1186/s12866-020-01977-3)
Supplement: Supplementary file 1 — Additional file 1: Table S1. Soil and plant factors of samples. Group name (E, R and S: years 1, 2, and 3, respectively; W, G and D: Glycyrrhiza uralensis, Glycyrrhiza glabra, and Glycyrrhiza inflata, respectively). Table S2. Sequencing results of each sample. Raw reads refers to the sequence filtering out low-quality bases; clean reads refers to the sequence finally used for subsequent analysis after filtering chimeras; base refers to the number of bases of final clean reads; Avglen refers to the average length of clean reads. Q20 refers to the percentage of bases whose quality value is greater than 20 (sequencing error rate is less than 1%); GC (%) refers to the content of GC bases in clean reads; effective (%) refers to the percentage of the number of clean reads and the number of raw reads. Sample name: E, R and S: years 1, 2, and 3, respectively; W, G and D: Glycyrrhiza uralensis, Glycyrrhiza glabra and Glycyrrhiza inflata, respectively; the third number representing the replicate number. Table S3. Composition information of dominant bacteria at each classification level. Others: The sum of the undefined and unannotated parts. Group name: E, R and S: years 1, 2, and 3, respectively; W, G and D: Glycyrrhiza uralensis, Glycyrrhiza glabra and Glycyrrhiza inflata, respectively; the third number representing the replicate number. Table S4. The alpha diversity indices in each group. Community richness was identified using the Chao1 and ACE estimator. Community diversity was identified using the Shannon and Simpson indexes. Sequencing depth was characterized by Good’s coverage, good’s coverage estimator values ranged from 99.6 to 99.8%, indicating that the sequence numbers per group were high enough. Group: E, R and S: years 1, 2, and 3, respectively; W, G and D: Glycyrrhiza uralensis, Glycyrrhiza glabra and Glycyrrhiza inflata, respectively. Table S5. Spearman correlation analyses testing the relationship between the Biomarker and the relationship between the physico [file 12866_2020_1977_MOESM1_ESM.docx]

Root-associated endophytic bacterial community composition and structure of three medicinal licorices and their changes with the growing year

**Hanli Dang ^1&^**, **Tao Zhang ^1&^, Guifang Li ^1^, Yudi Mu ^2^, Xinhua Lv ^1^ , Zhongke Wang ^1^ and Li Zhuang^1^***

^1^ College of Life Sciences, Key Laboratory of Xinjiang Phytomedicine Resource Utilization, Ministry of Education, Shihezi University, Xinjiang Shihezi, 832003, China.

^2^ 1300 UNIVERSITY Ave, MADISON, WI, 53706, USA.

^&^ These authors contributed equally to this work and should be considered co-first authors.

***** Correspondence and requests for materials should be addressed to Li Zhuang (E-mail: 3033573705@qq.com).

**Supplementary Table S1-6**

**Supplementary Table S1** soil and plant factors of samples

| **Group** |  | **E** | | | **R** | | | **S** | | |
| --- | --- | --- | --- | --- | --- | --- | --- | --- | --- | --- |
| **temperature** | Annual average temperature (°C) | 10.065 | | | 8.917 | | | 9.985 | | |
| **rainfall** | Annual rainfall(mm) | 46.9 | | | 65 | | | 23.2 | | |
|  |  | **W** | **D** | **G** | **W** | **D** | **G** | **W** | **D** | **G** |
| **Soil** | SOM (g/kg) | 5.713 | 3.912 | 3.806 | 5.517 | 3.912 | 6.131 | 4.628 | 5.850 | 4.028 |
|  | STN (g/kg) | 0.485 | 0.385 | 0.276 | 0.602 | 0.315 | 0.410 | 0.452 | 0.389 | 0.368 |
|  | STP (g/kg) | 0.403 | 0.512 | 0.389 | 0.622 | 0.401 | 0.471 | 0.465 | 0.409 | 0.428 |
|  | STK (g/kg) | 22.667 | 21.318 | 22.674 | 22.425 | 24.374 | 23.757 | 21.174 | 21.327 | 21.257 |
|  | SAN (mg/kg) | 3.572 | 3.703 | 3.857 | 7.434 | 5.007 | 6.209 | 5.844 | 5.490 | 5.217 |
|  | SNN (mg/kg) | 16.452 | 12.196 | 17.829 | 4.871 | 4.680 | 3.616 | 5.943 | 16.962 | 4.204 |
|  | TS (g/kg) | 10.283 | 4.660 | 10.075 | 0.875 | 1.450 | 1.792 | 4.842 | 4.342 | 2.075 |
| **plant** | POC (g/kg) | 418.281 | 359.010 | 406.051 | 394.485 | 384.198 | 431.368 | 402.430 | 404.794 | 323.093 |
|  | PWC (%) | 66.342 | 65.168 | 65.780 | 56.871 | 47.149 | 50.057 | 46.199 | 46.526 | 50.580 |
|  | PTN (g/kg) | 21.783 | 19.388 | 24.152 | 23.749 | 19.395 | 20.779 | 23.147 | 18.543 | 17.890 |
|  | PTP (g/kg) | 2.300 | 1.836 | 1.994 | 1.815 | 1.464 | 1.370 | 2.119 | 1.916 | 1.531 |
|  | PTK (g/kg) | 18.200 | 15.203 | 15.714 | 13.432 | 9.362 | 11.755 | 13.929 | 10.332 | 10.606 |
|  | CF (%) | 14.601 | 19.069 | 21.478 | 15.504 | 12.723 | 13.303 | 12.055 | 10.996 | 12.417 |

Description: group name (E, R and S: years 1, 2, and 3, respectively; W, G and D: *Glycyrrhiza uralensis*, *Glycyrrhiza glabra*, and *Glycyrrhiza inflata,* respectively).

**Supplementary Table S2** Sequencing results of each sample.

| **Sample name** | **Raw reads** | **Clean reads** | **Base(nt)** | **Avglen(nt)** | **Q20** | **GC %** | **Effective %** |
| --- | --- | --- | --- | --- | --- | --- | --- |
| E.W.1 | 52611 | 49096 | 18387028 | 374 | 86.06 | 54.63 | 93.32 |
| E.W.2 | 56921 | 51787 | 19399818 | 374 | 85.89 | 55.14 | 90.98 |
| E.W.3 | 87325 | 80087 | 29983882 | 374 | 84.9 | 55.42 | 91.71 |
| R.W.1 | 85245 | 80233 | 30010838 | 374 | 85.44 | 53.3 | 94.12 |
| R.W.2 | 75497 | 72973 | 27274486 | 373 | 86.28 | 54.63 | 96.66 |
| R.W.3 | 86828 | 80128 | 29936968 | 373 | 86.15 | 54.61 | 92.28 |
| S.W.1 | 85201 | 80136 | 29753364 | 371 | 83.12 | 55.26 | 94.06 |
| S.W.2 | 54803 | 53352 | 19790355 | 370 | 85.09 | 54.18 | 97.35 |
| S.W.3 | 82028 | 80204 | 29915432 | 372 | 84.15 | 55.28 | 97.78 |
| E.D.1 | 76482 | 73920 | 26909685 | 364 | 79.84 | 53.75 | 96.65 |
| E.D.2 | 82737 | 80173 | 29821914 | 371 | 85.19 | 54.21 | 96.9 |
| E.D.3 | 88466 | 80245 | 29661789 | 369 | 85.68 | 53.88 | 90.71 |
| R.D.1 | 83086 | 80156 | 29783243 | 371 | 85.06 | 55.22 | 96.47 |
| R.D.2 | 84279 | 80128 | 29914582 | 373 | 86.07 | 55.01 | 95.07 |
| R.D.3 | 86162 | 83753 | 31352290 | 374 | 85.85 | 55.51 | 97.2 |
| S.D.1 | 86486 | 80257 | 29850621 | 371 | 85.61 | 54.41 | 92.8 |
| S.D.2 | 60230 | 57509 | 21478388 | 373 | 84.49 | 54.43 | 95.48 |
| S.D.3 | 83885 | 80033 | 29932343 | 374 | 85.97 | 53.9 | 95.41 |
| E.G.1 | 85604 | 80093 | 29594733 | 369 | 82.84 | 54.82 | 93.56 |
| E.G.2 | 53890 | 50638 | 18543796 | 366 | 79.01 | 54.64 | 93.97 |
| E.G.3 | 82336 | 80121 | 29908902 | 373 | 84.95 | 55.12 | 97.31 |
| R.G.1 | 82689 | 80092 | 29944052 | 373 | 86.39 | 54.14 | 96.86 |
| R.G.2 | 78178 | 70880 | 26542711 | 374 | 85.59 | 54.36 | 90.66 |
| R.G.3 | 56302 | 53178 | 19878749 | 373 | 85.8 | 54.66 | 94.45 |
| S.G.1 | 82325 | 80201 | 30020819 | 374 | 85.63 | 54.67 | 97.42 |
| S.G.2 | 87833 | 80100 | 29486543 | 368 | 79.07 | 54.5 | 91.2 |
| S.G.3 | 87955 | 80058 | 29628594 | 370 | 84.15 | 55.57 | 91.02 |

Description: Raw reads refers to the sequence filtering out low-quality bases; clean reads refers to the sequence finally used for subsequent analysis after filtering chimeras; base refers to the number of bases of final clean reads; Avglen refers to the average length of clean reads. Q20 refers to the percentage of bases whose quality value is greater than 20 (sequencing error rate is less than 1%); GC (%) refers to the content of GC bases in clean reads; effective (%) refers to the percentage of the number of clean reads and the number of raw reads. Sample name: E, R and S: years 1, 2, and 3, respectively; W, G and D: *Glycyrrhiza uralensis*, *Glycyrrhiza glabra* and *Glycyrrhiza inflata*, respectively; the third number representing the replicate number.

**Supplementary Table S3** Composition information of dominant bacteria at each classification level

|  | **Taxonomy** |  | **W** |  |  | **D** |  |  | **G** |  |
| --- | --- | --- | --- | --- | --- | --- | --- | --- | --- | --- |
|  |  | **E** | **R** | **S** | **E** | **R** | **S** | **E** | **R** | **S** |
| **Class** | Gammaproteobacteria | 37.409% | 24.519% | 16.306% | 69.261% | 16.653% | 33.129% | 20.468% | 25.274% | 13.836% |
|  | Alphaproteobacteria | 41.900% | 38.608% | 62.456% | 13.093% | 47.275% | 53.946% | 41.822% | 48.389% | 68.278% |
|  | Bacteroidia | 3.414% | 19.711% | 6.819% | 7.781% | 4.442% | 4.916% | 5.642% | 7.031% | 2.052% |
|  | unidentified-Actinobacteria | 14.040% | 5.278% | 7.358% | 7.676% | 20.627% | 3.499% | 17.874% | 3.430% | 8.916% |
|  | Clostridia | 0.128% | 4.881% | 1.515% | 0.272% | 0.235% | 0.865% | 1.476% | 4.228% | 0.085% |
|  | unidentified-Bacteria | 0.039% | 0.710% | 0.231% | 0.115% | 4.993% | 0.172% | 0.531% | 0.081% | 0.344% |
|  | Bacilli | 1.144% | 1.387% | 1.149% | 0.591% | 0.223% | 1.377% | 5.973% | 4.486% | 0.660% |
|  | Deltaproteobacteria | 0.564% | 1.293% | 1.480% | 0.491% | 1.731% | 0.804% | 2.218% | 1.295% | 3.696% |
|  | unidentified-Chlamydiae | 0.116% | 1.707% | 0.070% | 0.134% | 0.845% | 0.151% | 0.192% | 0.133% | 0.415% |
|  | Mollicutes | 0.048% | 0.235% | 0.107% | 0.137% | 0.081% | 0.154% | 0.886% | 1.021% | 0.060% |
|  | Others | 1.198% | 1.672% | 2.510% | 0.450% | 2.895% | 0.987% | 2.918% | 4.632% | 1.659% |
| **Older** | Rhizobiales | 27.135% | 22.080% | 43.118% | 6.181% | 30.971% | 23.135% | 25.243% | 25.789% | 36.054% |
|  | Enterobacteriales | 1.287% | 1.285% | 1.637% | 36.206% | 1.064% | 10.291% | 3.787% | 2.186% | 1.257% |
|  | Sphingomonadales | 7.675% | 8.778% | 10.549% | 4.475% | 7.851% | 23.792% | 8.219% | 12.079% | 14.704% |
|  | Bacteroidales | 0.083% | 12.092% | 3.922% | 0.665% | 0.609% | 2.238% | 2.989% | 5.841% | 0.228% |
|  | Pseudomonadales | 15.286% | 6.486% | 4.765% | 14.328% | 3.879% | 16.691% | 2.963% | 8.034% | 3.436% |
|  | Oceanospirillales | 3.064% | 0.067% | 0.067% | 13.639% | 0.063% | 0.062% | 0.425% | 0.078% | 0.214% |
|  | Caulobacterales | 4.391% | 5.686% | 6.762% | 1.386% | 6.446% | 5.020% | 4.628% | 9.491% | 16.695% |
|  | unidentified-Gammaproteobacteria | 8.928% | 14.053% | 6.714% | 2.842% | 10.145% | 4.762% | 9.221% | 12.605% | 7.915% |
|  | Streptosporangiales | 7.549% | 0.008% | 0.011% | 0.190% | 0.008% | 0.004% | 0.186% | 0.016% | 0.005% |
|  | Flavobacteriales | 0.665% | 2.667% | 1.234% | 6.848% | 0.416% | 2.237% | 1.225% | 0.507% | 0.662% |
|  | Others | 23.935% | 26.798% | 21.222% | 13.239% | 38.549% | 11.769% | 41.115% | 23.374% | 18.831% |
| **Family** | Rhizobiaceae | 20.562% | 12.805% | 35.932% | 4.295% | 20.778% | 15.785% | 14.609% | 18.795% | 28.915% |
|  | Enterobacteriaceae | 1.287% | 1.285% | 1.637% | 36.206% | 1.064% | 10.291% | 3.787% | 2.186% | 1.257% |
|  | Sphingomonadaceae | 7.675% | 8.778% | 10.549% | 4.475% | 7.851% | 23.792% | 8.219% | 12.079% | 14.704% |
|  | Pseudomonadaceae | 15.195% | 6.342% | 4.662% | 7.165% | 3.836% | 16.584% | 2.538% | 7.772% | 3.346% |
|  | Halomonadaceae | 2.574% | 0.034% | 0.027% | 10.824% | 0.023% | 0.046% | 0.181% | 0.051% | 0.051% |
|  | Caulobacteraceae | 4.338% | 5.656% | 6.746% | 1.377% | 6.433% | 4.824% | 4.476% | 9.467% | 16.674% |
|  | Nocardiopsaceae | 7.545% | 0.008% | 0.011% | 0.182% | 0.003% | 0.001% | 0.134% | 0.004% | 0.005% |
|  | Bacteroidaceae | 0.011% | 5.493% | 1.517% | 0.290% | 0.261% | 0.946% | 1.529% | 2.781% | 0.066% |
|  | Flavobacteriaceae | 0.548% | 2.340% | 0.649% | 4.994% | 0.360% | 0.578% | 0.723% | 0.392% | 0.268% |
|  | Unidentified-Bacteria | 0.039% | 0.696% | 0.231% | 0.115% | 4.992% | 0.172% | 0.455% | 0.072% | 0.344% |
|  | Others | 40.226% | 56.564% | 38.039% | 30.076% | 54.399% | 26.981% | 63.349% | 46.403% | 34.370% |
| **Species** | *Pantoea-brenneri* | 0.146% | 0.149% | 0.112% | 14.362% | 0.068% | 1.914% | 0.642% | 0.132% | 0.173% |
|  | *Neorhizobium-huautlense* | 1.977% | 2.142% | 0.648% | 0.289% | 0.566% | 3.019% | 3.191% | 1.026% | 11.756% |
|  | *Pseudomonas-psychrotolerans* | 8.507% | 0.324% | 0.149% | 0.227% | 0.017% | 0.069% | 0.199% | 0.167% | 0.069% |
|  | *Halomonas-titanicae* | 0.975% | 0.005% | 0.009% | 3.551% | 0.007% | 0.005% | 0.062% | 0.004% | 0.007% |
|  | *Neorhizobium-alkalisoli* | 0.023% | 0.014% | 0.025% | 0.023% | 2.957% | 0.038% | 0.053% | 0.000% | 0.011% |
|  | *Salinicola-zeshunii* | 0.175% | 0.026% | 0.008% | 3.194% | 0.009% | 0.023% | 0.072% | 0.018% | 0.008% |
|  | *Nocardioides-albus* | 0.043% | 0.238% | 0.089% | 0.022% | 3.134% | 0.020% | 0.172% | 0.027% | 0.772% |
|  | *Promicromonospora-aerolata* | 0.792% | 0.414% | 1.012% | 1.189% | 3.012% | 0.231% | 1.911% | 0.176% | 0.102% |
|  | *bacterium-GWC2-44-17* | 0.004% | 0.502% | 0.043% | 0.017% | 2.307% | 0.023% | 0.166% | 0.030% | 0.005% |
|  | *Pseudomonas-indica* | 2.273% | 0.002% | 0.005% | 0.001% | 0.002% | 0.000% | 0.003% | 0.012% | 0.004% |
|  | Others | 85.084% | 96.184% | 97.901% | 77.126% | 87.921% | 94.659% | 93.529% | 98.407% | 87.093% |

Description: Others: The sum of the undefined and unannotated parts. Group name: E, R and S: years 1, 2, and 3, respectively; W, G and D: *Glycyrrhiza uralensis*, *Glycyrrhiza glabra* and *Glycyrrhiza inflata*, respectively; the third number representing the replicate number.

**Supplementary Table S4** The alpha diversity indices in each group

| **Group** | **Observed-species** | **Shannon** | **Simpson** | **Chao1** | **ACE** | **Goods-coverage** | **PD-Whole tree** |
| --- | --- | --- | --- | --- | --- | --- | --- |
|  |  | **diversity indexes** | | **richness indexes** | |  |  |
| **E.D** | 471 | 4.683 | 0.876 | 599.322 | 591.548 | 0.996 | 39.343 |
| **E.G** | 667 | 7.242 | 0.983 | 766.419 | 762.135 | 0.996 | 60.465 |
| **E.W** | 457 | 5.681 | 0.944 | 512.775 | 517.843 | 0.998 | 72.007 |
| **R.D** | 551 | 6.089 | 0.967 | 645.196 | 662.900 | 0.996 | 54.687 |
| **R.G** | 685 | 6.219 | 0.933 | 766.386 | 758.080 | 0.997 | 96.671 |
| **R.W** | 550 | 6.559 | 0.972 | 658.281 | 649.357 | 0.996 | 69.853 |
| **S.D** | 520 | 5.091 | 0.901 | 588.605 | 606.469 | 0.997 | 42.475 |
| **S.G** | 471 | 5.222 | 0.928 | 578.300 | 577.414 | 0.996 | 58.674 |
| **S.W** | 629 | 6.041 | 0.911 | 732.927 | 729.696 | 0.996 | 78.207 |

Description: Community richness was identified using the Chao1 and ACE estimator. Community diversity was identified using the Shannon and Simpson indexes. Sequencing depth was characterized by Good’s coverage, good’s coverage estimator values ranged from 99.6% to 99.8%, indicating that the sequence numbers per group were high enough. Group: E, R and S: years 1, 2, and 3, respectively; W, G and D: *Glycyrrhiza uralensis*, *Glycyrrhiza glabra* and *Glycyrrhiza inflata*, respectively.

**Supplementary Table S5** Spearman correlation analyses testing the relationship between the Biomarker and the relationship between the physicochemical properties of soil, leaf nutrients and bioactive compounds of licorice root.

|  | **Gammaproteobacteria** | **Oceanospirillales** | **Streptosporangiales** | **Halomonadaceae** | **Nocardiopsaceae** | ***Halomonas*** | ***Nocardiopsis*** | ***Sinomicrobium*** | ***Pelagibacterium*** | ***Methylophilus*** |
| --- | --- | --- | --- | --- | --- | --- | --- | --- | --- | --- |
| SOM | 0.086 | -0.426* | -0.061 | -0.35 | -0.084 | -0.277 | -0.084 | -0.114 | -0.344 | -0.075 |
| STN | 0.224 | -0.289 | -0.022 | -0.258 | 0.02 | -0.361 | 0.02 | 0.038 | -0.12 | 0.067 |
| STP | 0.209 | -0.169 | 0.04 | -0.12 | 0.098 | -0.304 | 0.098 | 0.131 | -0.162 | -0.117 |
| STK | 0.156 | -0.248 | 0.073 | -0.127 | 0.018 | -0.138 | 0.018 | -0.006 | -0.083 | -0.003 |
| SNN | 0.373 | 0.207 | 0.395* | 0.28 | 0.302 | 0.384* | 0.302 | 0.397* | 0.356 | 0.148 |
| SAN | -0.223 | -0.540** | -0.590** | -0.556** | -0.58** | –0.577** | -0.581** | -0.136 | -0.641** | 0.024 |
| TS | 0.146 | 0.506** | 0.484* | 0.537** | 0.448* | 0.648** | 0.448* | 0.378 | 0.672** | 0.131 |
| PWC | 0.513** | 0.444* | 0.592** | 0.503** | 0.684** | 0.557** | 0.684** | 0.499** | 0.427 | 0.366 |
| POC | 0.221 | -0.108 | 0.159 | 0.152 | 0.064 | 0.116 | 0.064 | 0.143 | 0.248* | 0.308 |
| PTN | -0.001 | -0.045 | 0.233 | 0.055 | 0.226 | -0.077 | 0.226 | 0.141 | 0.25 | 0.319 |
| PTP | 0.281 | 0.323 | 0.454* | 0.412* | 0.444* | 0.378 | 0.444* | 0.211 | 0.530** | 0.305 |
| PTK | 0.469* | 0.571** | 0.739** | 0.667** | 0.760** | 0.640** | 0.760** | 0.496** | 0.769** | 0.305 |
| CF | 0.300 | 0.384* | 0.657** | 0.471* | 0.729** | 0.404* | 0.729** | 0.578** | 0.408* | 0.272 |
| GlA | -0.399* | -0.610** | -0.705** | -0.663** | -0.689** | -0.670** | -0.689** | -0.491** | -0.673** | -0.239 |
| GTF | -0.623** | -0.558** | -0.583** | -0.569** | -0.634** | -0.532** | -0.634** | -0.390* | -0.575** | -0.159 |
| LI | -0.416* | -0.616** | -0.695** | -0.657** | -0.686** | -0.674** | -0.686** | -0.508** | -0.694** | -0.275 |

Description: The values are the correlation coefficients. ** means *P*<0.01; * means *P*<0.05.

**Supplementary Table S6** Correlations between environmental factors and endophytic bacterial communities in each group.

| **Variable** | ***r*** | ***P* Value** |
| --- | --- | --- |
| SOM+STN+STP+STK+SNN+SAN+TS | 0.123 | 0.136 |
| GlA+ GTF+LI | 0.260 | 0.008 |
| POC+PTN+PTP+PTK+CF+PWC | 0.042 | 0.290 |
| STN+STP+STK+PTN+PTP+PTK | 0.133 | 0.081 |
| SOM+SNN+SAN+TS | 0.092 | 0.204 |
| CF+POC+PWC | 0.010 | 0.468 |
| GlA+ GTF+LI+STN+STP+STK+PTN+PTP+PTK | 0.238 | 0.010 |
| GlA+ GTF+LI+SOM+SNN+SAN+TS | 0.197 | 0.041 |
| GlA+ GTF+LI+CF+POC+PWC | 0.178 | 0.028 |

Description: Variable is the information of environmental factors, *r* is the correlation coefficient, and *P* value is the *p*-value of significance test. The larger *r* value is, the greater the correlation between environmental factors and species abundance information is. *P*<0.05 indicates statistical significance.
